# HIF1α-AS1 is a DNA:DNA:RNA triplex-forming lncRNA interacting with the HUSH complex

**DOI:** 10.1038/s41467-022-34252-2

**Published:** 2022-11-02

**Authors:** Matthias S. Leisegang, Jasleen Kaur Bains, Sandra Seredinski, James A. Oo, Nina M. Krause, Chao-Chung Kuo, Stefan Günther, Nevcin Sentürk Cetin, Timothy Warwick, Can Cao, Frederike Boos, Judit Izquierdo Ponce, Shaza Haydar, Rebecca Bednarz, Chanil Valasarajan, Dominik C. Fuhrmann, Jens Preussner, Mario Looso, Soni S. Pullamsetti, Marcel H. Schulz, Hendrik R. A. Jonker, Christian Richter, Flávia Rezende, Ralf Gilsbach, Beatrice Pflüger-Müller, Ilka Wittig, Ingrid Grummt, Teodora Ribarska, Ivan G. Costa, Harald Schwalbe, Ralf P. Brandes

**Affiliations:** 1 Institute for Cardiovascular Physiology, Goethe University, Frankfurt, Germany; 2 German Center of Cardiovascular Research (DZHK), Partner site RheinMain, Frankfurt, Germany; 3 Institute for Organic Chemistry and Chemical Biology, Center for Biomolecular Magnetic Resonance (BMRZ), Goethe University, Frankfurt, Germany; 4 Institute for Computational Genomics, Joint Research Center for Computational Biomedicine, RWTH Aachen Medical Faculty, Aachen, Germany; 5 Max Planck Institute for Heart and Lung Research, Bad Nauheim, Germany; 6 Division of Molecular Biology of the Cell II, German Cancer Research Center DKFZ-ZMBH, Heidelberg, Germany; 7 Department of Internal Medicine, Member of the DZL, Member of Cardio-Pulmonary Institute (CPI), Justus Liebig University, Gießen, Germany; 8 Faculty of Medicine, Institute of Biochemistry I, Goethe University, Frankfurt, Germany; 9 Institute of Cardiovascular Regeneration, Goethe University, Frankfurt, Germany; 10 Functional Proteomics, SFB 815 Core Unit, Faculty of Medicine, Goethe University, Frankfurt, Germany; 11 Oslo University Hospital, Oslo, Norway

**Keywords:** Cardiovascular biology, Nucleic acids, Long non-coding RNAs

## Abstract

DNA:DNA:RNA triplexes that are formed through Hoogsteen base-pairing of the RNA in the major groove of the DNA duplex have been observed in vitro, but the extent to which these interactions occur in cells and how they impact cellular functions remains elusive. Using a combination of bioinformatic techniques, RNA/DNA pulldown and biophysical studies, we set out to identify functionally important DNA:DNA:RNA triplex-forming long non-coding RNAs (lncRNA) in human endothelial cells. The lncRNA *HIF1α-AS1* was retrieved as a top hit. Endogenous *HIF1α-AS1* reduces the expression of numerous genes, including EPH Receptor A2 and Adrenomedullin through DNA:DNA:RNA triplex formation by acting as an adapter for the repressive human silencing hub complex (HUSH). Moreover, the oxygen-sensitive *HIF1α-AS1* is down-regulated in pulmonary hypertension and loss-of-function approaches not only result in gene de-repression but also enhance angiogenic capacity. As exemplified here with *HIF1α-AS1*, DNA:DNA:RNA triplex formation is a functionally important mechanism of trans-acting gene expression control.

## Abstract (web summary)

Using a composite bioinformatics approach, the DNA:DNA:RNA triplex-forming lncRNAs HIF1α-AS1 was identified in human endothelial cells which recruits an epigenetic silencing complex to limit expression of triplex target genes.

## Introduction

Long non-coding RNAs (lncRNAs) represent the most diverse, plastic and poorly understood class of ncRNA^1^. Their gene regulatory mechanisms involve formation of RNA-protein, RNA-RNA or RNA-DNA complexes^1^. RNA-DNA interactions occur either in heteroduplex (DNA:RNA) or triplex strands (DNA:DNA:RNA). In triplexes, double-stranded DNA (dsDNA) accommodates the single-stranded RNA in its major groove^2^. The binding occurs via Hoogsteen or reverse Hoogsteen hydrogen bonds with a purine-rich sequence of DNA to which the RNA strand binds in a parallel or antiparallel manner. Hoogsteen bonds are weaker than Watson-Crick bonds, resulting in Hoogsteen pairing rules being more flexible^3^.

Ex vivo characterization of triplex formation relies on a variety of different biophysical methods including circular dichroism- (CD) and nuclear magnetic resonance-spectroscopy (NMR)^4–6^. Even with these techniques it can be challenging to discriminate DNA-RNA heteroduplexes from triplexes and analyses are usually restricted to oligonucleotides of a limited length. Nevertheless, a few lncRNAs have been suggested to form triplexes with dsDNA, however, triplex studies using living cells are still in early development^4,6–13^. In silico analyses of RNA-DNA triplex formation predicted several genomic loci and lncRNAs to form triplexes^14^. In line with this, a global approach in HeLa S3 and U2OS cells to isolate triplex-forming RNAs on a genome-wide scale yielded several RNA:DNA triplex-forming lncRNAs^15^.

In addition to the sparse initial findings of triplex formation within cells, several other open questions remain: What is the physiological relevance of triplex-forming lncRNAs and are these cell- and tissue-type specific? What is the mechanism of action of triplex-forming lncRNAs? Do they disturb transcription in a similar way to R-loops^16^ or recruit certain protein complexes to DNA in a site-specific manner? Regarding the latter aspect, Polycomb Repressive Complex 2 (PRC2) has been identified as a target of the lncRNAs HOX Transcript Antisense RNA (*HOTAIR*), FOXF1 Adjacent Non-Coding Developmental Regulatory RNA (*FENDRR*) and Maternally Expressed 3 (*MEG3*)^4,12,13^, but, given the highly promiscuous nature of PRC2, this function remains controversial. Other examples of protein interactors involve e.g. E2F1 and p300, which are recruited by the triplex-forming antisense lncRNA *KHPS1* to activate gene expression of the proto-oncogene sphingosine kinase 1 (SPHK1) *in cis*^7,10^.

Much of today’s in vitro RNA research heavily relies on immortalized cell lines. Although such model systems are well suited for transfection or genomic manipulation, they are highly de-differentiated and exhibit reaction patterns such as unlimited growth and immortalization - characteristics not observed in primary cells^17^. Considering that lncRNAs are expressed in a species-, tissue- and differentiation-specific manner^1^, biological evidence for lncRNA functions in primary cells is limited. Among such cells, endothelial cells stand out due to their well documented importance in regeneration, angiogenesis and tissue vascularization. Indeed, endothelial cell dysfunction is one of the main drivers of systemic diseases like diabetes and inflammation^18^.

Here, we combined molecular biology and biophysics, bioinformatics and physiology to systematically uncover the role of triplex-forming lncRNAs in endothelial cells. This approach identified *HIF1α-AS1* as a *trans*-acting triplex-forming lncRNA that controls vascular gene expression in endothelial cells with implications for vascular disease.

## Results

### HIF1α-AS1 is a triplex-associated lncRNA

**Fig. 1:**
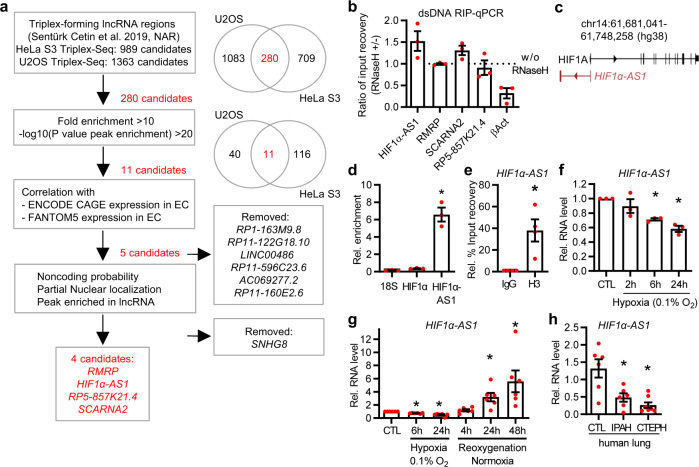
*HIF1α-AS1* is a triplex- and DNA-associated RNase H-insensitive lncRNA in endothelial cells. **a** Overview of the identification of endothelial-expressed triplex-forming lncRNAs. LncRNAs from a previous Triplex-Seq study in HeLa S3 and U2OS were overlapped, filtered with high stringency and analyzed for nuclear expression in endothelial cells with ENCODE and FANTOM5 CAGE data followed by analyses for noncoding probability and enriched peaks in the Triplex-Seq data. **b** RNA-immunoprecipitation with anti-dsDNA antibody followed by qPCR (RIP-qPCR) targeting the lncRNA candidates in HUVEC. Samples were treated with or without RNase H. βAct served as control for RNase H-mediated degradation. *n* = 3. **c** Scheme of the human genomic locus of *HIF1α-AS1*. **d** RT-qPCR after anti-dsDNA-RIP in HUVEC. *HIF1α* and *18* *S* rRNA served as negative control. One-way ANOVA with Tukey’s test, *n* = 3. *(*p* = 0.0002). **e** RIP-qPCR with anti-histone3 (H3) in HUVEC. Data was normalized against GAPDH. Paired *t* test, *n* = 4. *(*p* = 0.0364). **f** RT-qPCR of *HIF1α-AS1* in HUVEC treated with hypoxia (0.1% O_2_) for the indicated time points. Normoxia served as negative control (CTL). *n* = 3, One-Way ANOVA with Bonferroni test. *6 h (*p* = 0.0216), * 24 h (*p* = 0.0035). **g** RT-qPCR of *HIF1α-AS1* in HUVECs treated with hypoxia (0.1% O_2_) followed by reoxygenation with normoxia (after 24 h of hypoxia) for the indicated time points. *n* = 6, paired *t* test. *Hypoxia-6h (*p* = 0.0001), *Hypoxia-24h (*p* = 0.002), *Reoxygenation-24h (*p* = 0.0189), *Reoxygenation-48h (*p* = 0.04). **h** RT-qPCR of *HIF1α-AS1* in lungs from control donors (CTL, *n* = 6) or patients with idiopathic pulmonary arterial hypertension (IPAH, *n* = 6) or chronic thromboembolic pulmonary hypertension (CTEPH, *n* = 8). One-Way ANOVA with Tukey’s test. *IPAH (*p* = 0.0063), *CTEPH (*p* = 0.0005). For **b**, **d**–**g**, *n* is defined as number of independent experiments. Data are presented as mean values ± SEM.

To identify triplex-associated lncRNAs, we used Triplex-Seq data from U2OS and HeLa S3 cells^15^. Triplex-Seq relies on the isolation of RNase H-resistant RNA-DNA complexes from cells followed by DNA- and RNA-Seq^15^. RNase H cleaves the RNA in DNA-RNA heteroduplexes as present in R-loops^19^ and has previously been used to distinguish between heteroduplexes and triplexes^20^. The Triplex-Seq data comprised all RNA entities and was filtered for the number of individual lncRNA genes, resulting in 989 (for HeLa S3, Supplementary Data 1) and 1363 (for U2OS, Supplementary Data 2) different lncRNAs associated with triplexes, with an overlap of 280 lncRNA genes between the two cell lines (Fig. 1a). To further narrow down this set of enriched triplex-associated lncRNAs, parameters for specificity (fold enrichment >10, -log10(P value peak enrichment)) were increased so that 11 lncRNA candidates with high confidence remained. Subsequently, these were correlated to ENCODE and FANTOM5 Cap Analysis of Gene Expression (CAGE)^21–23^ data. Of the 11 candidates, only 5 (*RMRP*, *HIF1α-AS1*, *RP5-857K21.4*, *SCARNA2* and *SNHG8*) were expressed in endothelial cells. All 5 candidates were predicted as non-coding by the online tools Coding Potential Assessment Tool (CPAT 3.0.0) and coding potential calculator 2 (CPC2) and at least partially nuclear localized by ENCODE CAGE (Fig. 1a). To further analyze these candidates, the Triplex-Seq enriched regions were manually inspected in the IGV browser. This led to the exclusion of *SNHG8* as the triplex-associated regions within this lncRNA were exclusively within the overlapping small nucleolar RNA 24 (*SNORA24*) gene. In the case of the other candidates, triplex-association was within the individual lncRNA gene body. The cumulative fold enrichment of the remaining lncRNAs in the Triplex-Seq dataset illustrated strong triplex-association (Supplementary Fig. 1a). To verify the candidates experimentally, RNA immunoprecipitation (RIP) with antibodies against dsDNA with or without RNase H treatment in human endothelial cells was performed. Cleavage of the RNA in DNA-RNA heteroduplexes by RNase H^19^ revealed that *HIF1α-AS1* was the strongest triplex-associated lncRNA (Fig. 1b).

Genomically, *HIF1α-AS1* is located on the antisense strand of the Hypoxia-inducible factor 1-alpha gene (*HIF1A*) (Fig. 1c). The lncRNA was specifically enriched in nuclear DNA, whereas *HIF1α* mRNA and *18* *S* rRNA were not (Fig. 1d). Moreover, RIP with anti-histone 3 (Fig. 1e) indicated that *HIF1α-AS1* is bound to dsDNA in the chromatin environment.

### HIF1α-AS1 is disease-relevant

Only a few studies have so far documented the biological relevance of *HIF1α-AS1*. Increased *HIF1α-AS1* expression has been reported in thoracoabdominal aortic aneurysms^24^. *HIF1α-AS1* was also suggested as a biomarker in colorectal carcinoma^25^. Functionally, *HIF1α-AS1* is pro-apoptotic and anti-proliferative in vascular smooth muscle, Kupffer and umbilical vein endothelial cells^26–28^.

As HIF1α is a central regulator of oxygen-dependent gene expression^18^, we decided to measure the expression of *HIF1α-AS1* in endothelial cells under altered oxygen and disease conditions. Hypoxia led to a decrease in *HIF1α-AS1* expression in endothelial and pulmonary artery smooth muscle cells (paSMC) (Fig. 1f, Supplementary Fig. 1b), which was restored in endothelial cells after 4 h and even surpassed basal levels after 24 h of normoxic conditions (Fig. 1g). Importantly, *HIF1α-AS1* was downregulated in endothelial cells isolated from human glioblastoma (Supplementary Fig. 1c) and in lungs from patients with end stage idiopathic pulmonary arterial hypertension (IPAH) or chronic thromboembolic pulmonary hypertension (CTEPH) (Fig. 1h). In paSMCs isolated from pulmonary arteries of patients with IPAH, *HIF1α-AS1* was strongly decreased (Supplementary Fig. 1d). Together, these data demonstrate that *HIF1α-AS1* is an oxygen-dependent and disease-relevant lncRNA.

### HIF1α-AS1-triplex binding suppresses target gene expression

**Fig. 2:**
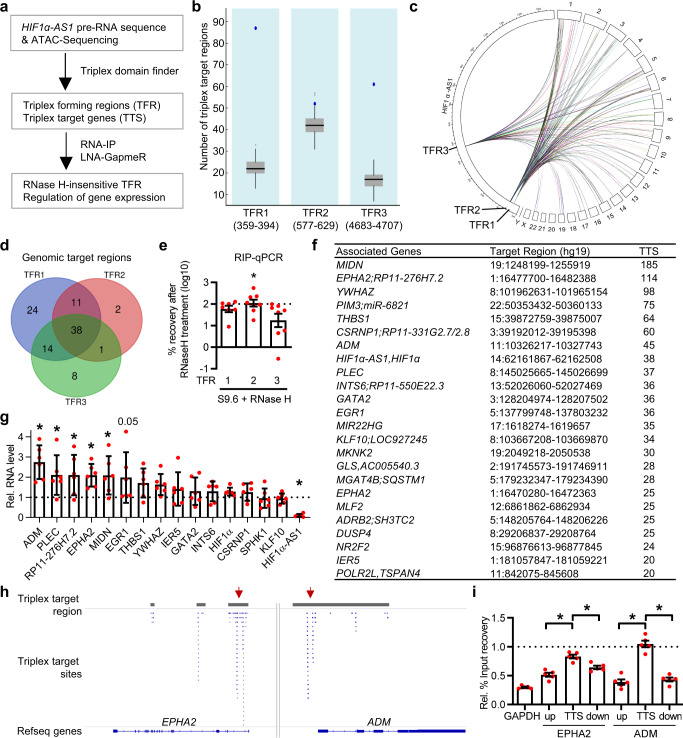
*HIF1α-AS1* potentially forms DNA:DNA:RNA triplexes. **a** Overview of the identification of *HIF1α-AS1* triplex forming regions (TFR) and their DNA triplex target sites (TTS) with triplex domain finder (TDF). *HIF1α-AS1* pre-RNA and ATAC-Seq of HUVECs treated with or without LNA GapmeRs against *HIF1α-AS1* were used as input for TDF. RIP and LNA GapmeRs were used to validate the findings of TDF. **b** All three TFRs of *HIF1α-AS1* have a significantly higher number of DNA triplex target regions (blue dots) than the random background (boxplots in gray). Boxplot visualizes the median, first and third quartiles. The whiskers present the 1.5 interquartile range. External gray dots represent outliers. Numbers in brackets are positions of the individual TFR within *HIF1α-AS1* pre-RNA. Analyzed with TDF, n=200 randomizations. **c** Circos plot showing the localization of the individual TFR within *HIF1α-AS1* pre-RNA and its interaction with the chromosomal TTS. **d** Overlap of TTS of the individual TFRs of *HIF1α-AS1*. **e** Identification of RNase H-resistant TFRs. RIP with anti-S9.6 with/without RNase H treatment in HUVEC followed by qPCR for the TFRs. Ratio of %-input recovery with/without RNase H treatment is shown. *n* = 8 independent experiments, paired *t* test. Dotted line represents normalized values without RNase H treatment. The asterisk indicates that the %-recovery after RNase H is significantly different for TFR2 compared to TFR1 (*p* = 0.0452) and TFR3 (*p* = 0.0244). **f** *HIF1α-AS1* TFR1-3 overlapping top target genes, their genomic location and number of TTS identified by TDF. **g** RT-qPCR of triplex target genes of TFR2 after knockdown of *HIF1α-AS1* in HUVEC. *n* = 6 independent experiments. One-Way ANOVA with Holm’s Sidak test. *ADM (*p* = <0.0001), *HIF1α-AS1 (*p* = <0.0001), *PLEC (*p* = 0.0238), *RP11-276H7.2 (*p* = 0.0238), *EPHA2 (*p* = 0.0238), *MIDN (*p* = 0.023). **h** Different triplex target regions of *HIF1α-AS1* are shown. Triplex target regions are highlighted in gray, triplex target sites are shown in blue. Arrows indicate TTS at the 5ʹend. **i** ChIP-qPCR with S9.6 antibody with/without RNase H treatment. Dotted line represents normalized values without RNase H. QPCR was performed against *EPHA2* or *ADM* TTS or regions up- and downstream of the individual TTS. One-Way ANOVA with Bonferonni test. *n* = 5 independent experiments. *EPHA2:up/TTS (*p* = <0.0001), *ADM:up/TTS (*p* = <0.0001), *ADM:TTS/down (*p* = <0.0001), *EPHA2:TTS/down (*p* = 0.0321). Data are presented as mean values ± SEM.

Triplex-Seq can provide evidence for existing triplex forming regions of the RNA (TFR) and triplex target sites (TTS) within the DNA but the details of exactly which TFR and TTS interact cannot be derived from Triplex-Seq. To identify the TFRs within *HIF1α-AS1* as well as *HIF1α-AS1*-dependent TTS, a combination of bioinformatics and wet lab approaches were used: An Assay for Transposase-Accessible Chromatin with high-throughput sequencing (ATAC-Seq) was performed after *HIF1α-AS1* knockdown to identify DNA target sites in human endothelial cells. LNA-GapmeRs targeting *HIF1α-AS1* led to a strong knockdown of the lncRNA (Supplementary Fig. 1e). Triplex Domain Finder (TDF), a computational tool for the prediction of RNA and DNA triplex-forming potential^14^, predicted the TFRs within *HIF1α-AS1* to target DNA regions around genes that displayed altered ATAC-Seq peaks after *HIF1α-AS1* silencing (Fig. 2a). The software identified three statistically significant TFRs (TFR1-3) within the pre-processed *HIF1α-AS1* RNA (Fig. 2b). There was also a high incidence of triplex-prone motifs predicted in regions whose chromatin state was altered in the ATAC-Seq data after *HIF1α-AS1* knockdown (Fig. 2c, Supplementary Data 3–5). Of these TTS, 38 overlapped within all three TFRs (Fig. 2d). To identify which TFR is most strongly associated with triplexes, RIP with S9.6 antibodies recognizing RNA-DNA association was performed. RNA-DNA associations remaining after RNase H treatment excluded the possibility that these were RNA-DNA heteroduplexes. Of the three *HIF1α-AS1* TFRs, TFR2 was identified as the TFR most resistant to RNase H (Fig. 2e). TFR2 is located intronically 478 nucleotides (nt) downstream of Exon1 and was detected by RT-PCR within nuclear isolated RNA with primers covering the first 714 nt (E1-I) of the pre-processed *HIF1α-AS1* (Supplementary Fig. 1f). Triplex-prone motifs in the TFR1-3-overlapping target regions yielded more than 20 different associated genes, some of which displayed a high number of DNA binding sites (Fig. 2f). If this binding of the lncRNA is relevant for the individual target gene, then a change in target gene expression would be expected. Importantly, in response to the downregulation of *HIF1α-AS1* with LNA-GapmeRs the expression of the following triplex target genes increased: *ADM*, *PLEC*, *RP11-276H7.2*, *EPHA2*, *MIDN* and *EGR1* (Fig. 2g). Interestingly, as exemplified by the target genes *HIF1A*, *EPHA2* and *ADM*, the triplex target sites are often located close to the 5ʹ end of the gene. In this region, histone modifications, transcription factor binding and chromatin conformation often have the greatest effect on promoter function and gene expression (Fig. 2h, Supplementary Fig. 1g). In order to prove that the triplexes also exist in vivo, Chromatin Immunoprecipitation (ChIP) was performed with S9.6 antibodies. After RNase H treatment, the TTS of *EPHA2* and *ADM* were both more resistent to RNase H treatment compared to DNA regions upstream or downstream of both TTS (Fig. 2i).

These data indicate that *HIF1α-AS1* contains triplex forming regions and target sites important for the regulation of gene expression.

### HIF1α-AS1 TFR2 RNA forms triplexes with EPHA2 and ADM

**Fig. 3:**
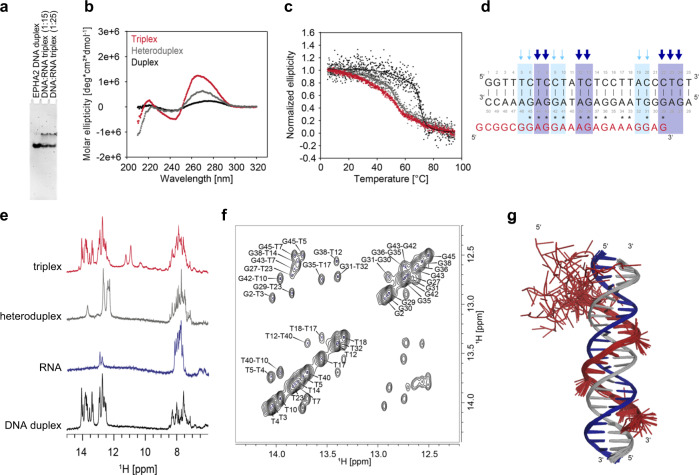
*HIF1α-AS1* TFR2 RNA forms in vitro DNA:DNA:RNA triplexes with the predicted DNA target region in *EPHA2*. **a** Electromobility shift assay of *EPHA2* DNA duplex and *EPHA2:HIF1α-AS1*_TFR2 triplex with 15 and 25 equivalents of RNA (TFO2-23). **b** Circular dichroism spectra of the *EPHA2* DNA duplex (black), the heteroduplex (dark gray) and *EPHA2:HIF1α-AS1*-TFR2 (red) measured at 298 K. **c** Thermal melting assay of the *EPHA2* DNA duplex (black), the heteroduplex (dark gray) and *EPHA2:HIF1α-AS1*-TFR2 (red). **d** Sequence of *EPHA2* DNA (black) and *HIF1α-AS1*-TFR2 RNA (red). Watson-Crick base pairing is indicated with | and the Hoogsteen base pairing is indicated with ^*^. Changes in the DNA duplex were quantitatively analyzed using NOESY spectra of duplex and triplex. Imino protons with strong attenuation (dark blue arrows) or medium attenuation (light blue arrows) of cross peak intensities in the imino-imino region were observed. **e** ^1^H-1D NMR spectra of the *EPHA2* DNA duplex (black), *HIF1α-AS1* TFR2 RNA (blue), heteroduplex (dark gray) and *EPHA2:HIF1α-AS1*-TFR2 triplex (red) at 288 K. **f** Assignment of the imino region of the ^1^H,^1^H-NOESY spectrum of *EPHA2* DNA duplex measured at 800 MHz and 288 K in NMR buffer with 5% D_2_O. **g** Cartoon representation of DNA:DNA:RNA triplex docking studies with the following color code: DNA strand (blue and gray) and RNA strand (red). This shows an ensemble of the 20 top-ranked modeled structures for a DNA:DNA:RNA triplex. The figure was generated by using PyMol 2.5 (Schrödinger, LLC). Source data (for a) are provided as a Source Data file.

Our analysis identified *HIF1α-AS1* TFR2 as the best suited candidate for verification of triplex formation of the lncRNA using biophysical and biochemical techniques. To monitor triplex formation of *HIF1α-AS1*, *EPHA2* was chosen as the target gene due to its abundance of triplex target sites (Figs. 2f, h), its regulatory potential (Fig. 2g) and its importance for vascularization^29^. Triplex domain finder predicted not the complete TFR2 to bind *EPHA2* TTS, but rather a core TFR2 sequence that binds the TTS. The formation of DNA:DNA:RNA triplexes between lncRNA *HIF1α-AS1* TFR2 and its proposed DNA target site within intron 1 of *EPHA2* was characterized by electrophoretic mobility shift assay (EMSA), CD- and solution NMR-spectroscopy. From electrophoretic mobility shift assay (EMSA) the *HIF1α-AS1* TFR2 RNA was found to form a low-mobility DNA-RNA complex with the *EPHA2* DNA target sequence (Fig. 3a). We also used CD-spectroscopy to confirm triplex formation of *HIF1α-AS1* TFR2 on *EPHA2*. The CD spectrum indicated typical features for triplex formation, such as a positive small peak at ∼220 nm, two negative peaks at ∼210 nm and ∼240 nm and a blue-shift of the peak at ∼270 nm^30,31^, which was distinct from the *EPHA2* DNA duplex or the heteroduplex spectra (Fig. 3b). This confirmed the existence of *EPHA2:HIF1α-AS1* TFR2 triplexes. Additionally, we performed thermal melting assays and obtained melting temperatures T_m_ (RNA-DNA heteroduplex) = 53.48 ± 0.32 °C, T_m_ (DNA-DNA duplex) = 70.74 ± 0.22 °C and T_m_ (DNA-DNA-RNA triplex) = 49.52 ± 0.22 °C with a very broad second melting point around 70 °C. The biphasic melting transition is a distinct feature of triplex formation, which is characterized by a first melting temperature that corresponds to melting of Hoogsteen hydrogen bonds that stabilize the triplex and the second for the melting of the Watson-Crick base pairing at higher temperatures (Fig. 3c). ^1^H-1D NMR spectra were recorded for *EPHA2* DNA duplex (25 nt), *HIF1α-AS1* TFR2 RNA (TFO2-23, 23 nt), *EPHA2:HIF1α-AS1*_TFR2 heteroduplex and *EPHA2:HIF1α-AS1*_TFR2 triplex at different temperatures (Fig. 3d). Using 10 eq *HIF1α-AS1* TFR2 RNA, triplex ^1^H NMR imino signals were observed in a spectral region between 9 and 12 ppm providing further evidence that *HIF1α-AS1* was associated with *EPHA2* through Hoogsteen base pairing (Fig. 3e, Supplementary Fig. 2a). Further, we conducted NMR-spectroscopic analysis of the triplex: we first measured a ^1^H, ^1^H-NOESY spectrum for *EPHA2* DNA duplex and assigned cross peaks in this spectrum of the DNA duplex. We identified 11 G and 12 T imino proton signals (Fig. 3f). Then, we added the *HIF1α-AS1*_TFR2 RNA triplex-forming strand in 10-fold excess [DNA-DNA]:[RNA] = 1:10 and semi-quantitatively analyzed the change in the DNA duplex spectrum. For 7 G- and 6 T-imino protons either a strong or medium attenuation of cross peak intensities in the imino-imino region of the NOESY spectrum was observed (Supplementary Fig. 2b). We rationalize this attenuation as to arise from weakening of the Watson-Crick base pairing induced by the Hoogsteen interaction with the RNA strand. From this analysis, we compared predicted Hoogsteen interactions in the triplex with the detected changes in the NOESY spectrum for different positions i of RNA relative to DNA duplex strand. Interestingly, the previously predicted position (i = 0) is supported by the observed attenuations in the NOESY, where 3 G- and 2 T-imino sites disappear completely and 3 G- and 1 T-imino sites are significantly attenuated. In total, 12 sites remain unaffected in the DNA duplex (Supplementary Fig. 2c). Further, based on our interpretation that Hoogsteen interactions can be mapped from the analysis of cross peak attenuation in the NOESY, we generated structural models for the *EPHA2:HIF1α-AS1*_TFR2 triplex. The ensemble of the 20 top-ranked structures for the triplex are displayed as cartoon (Fig. 3g).

To confirm the formation of triplexes with lower equivalents, stabilized triplex formation was investigated: the intermolecular dsDNA formed by two complementary antiparallel DNA strands was changed into a hairpin construct, where both DNA strands were linked with a 5 nt thymidine-linker and duplex formation thus became intramolecular. With this approach, triplex formation was obtained with 3 eq RNA, indicating that triplex formation is favored under those conditions as expected. ^1^H-1D NMR spectra of hairpin *EPHA2*_CTGA and ^15^N-labeled *HIF1α-AS1* TFR2:*EPHA2*_CTGA triplex indicated changes in the Hoogsteen region (9-12 ppm) and the spectral region of imino (12-14 ppm) and amino signals (7–8.5 ppm) (Supplementary Fig. 3a, b). In addition to *EPHA2*, we also tested *ADM*, a preprohormone involved in endothelial cell function^32^. For *ADM*_CTGA:*HIF1α-AS1* TFR2 triplex, the new imino protons in the Hoogsteen region arose at lower temperatures (Supplementary Fig. 3c and d). For both *ADM*_CTGA and *EPHA2*_CTGA triplex constructs the CD spectra showed an increased negative ellipticity at ∼240 nm and positive ellipticity at ∼270 nm (Supplementary Fig. 3e, f). Further, the thermal melting data verified the triplex stabilization with higher melting temperatures and defined melting transitions upon DNA hairpin formation. For the *EPHA2*_CTGA:*HIF1α-AS1* TFR2 (TFO2-23) triplex we obtained a first melting point at T_m_ (1^st^ triplex) = 50.08 ± 0.51 °C, a second melting point T_m_ (2^nd^ triplex) = 79.90 ± 0.10 °C and T_m_ (DNA hairpin) = 80.41 ± 0.10 °C (Supplementary Fig. 3g). The melting temperature of *ADM* DNA duplex T_m_ (DNA-DNA duplex) = 63.80 ± 0.20 °C increased for the *ADM*_CTGA hairpin T_m_ (DNA hairpin) = 95.76 ± 16.69 °C. For the *ADM*_CTGA:*HIF1α-AS1* TFR2 (TFO2-23), we obtained a first melting point T_m_ (1^st^ triplex) = 40.24 ± 2.62 °C and a second T_m_ (2^nd^ triplex) = 81.78 ± 0.59 °C (Supplementary Fig. 3h). The data demonstrate that *HIF1α-AS1* TFR2 forms triplexes with *EPHA2* and *ADM* dsDNA under regular and triplex-stabilized conditions upon DNA hairpin formation.

### TFR2 represses EPHA2 and ADM gene expression

**Fig. 4:**
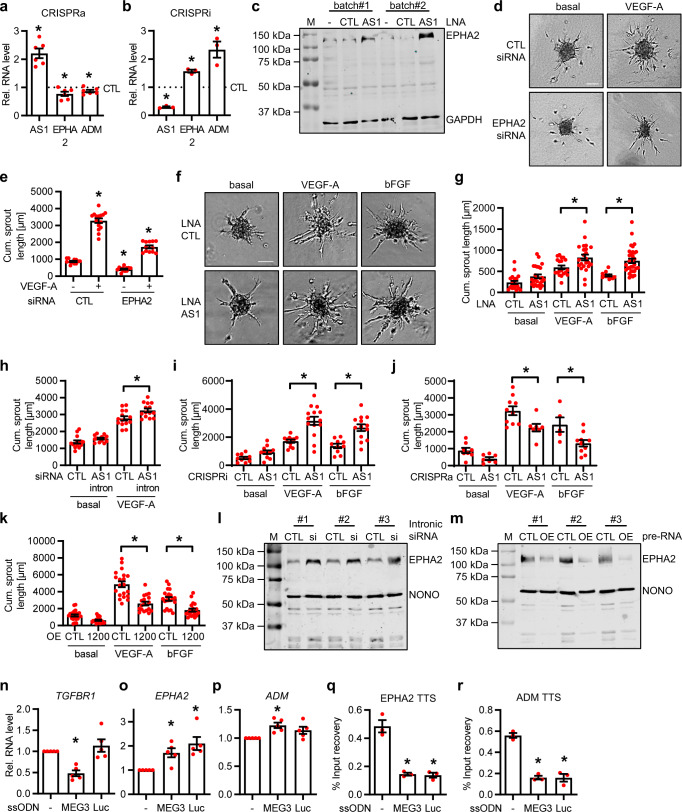
*HIF1α-AS1* limits EPHA2 and ADM expression through TFR2. **a**, **b** CRISPRa (**a**, *n* = 6 independent experiments) or CRISPRi (**b**, *n* = 3 independent experiments) targeting *HIF1α-AS1* in HUVECs followed by RT-qPCR for *HIF1α-AS1*, *EPHA2* and *ADM*. Paired *t* test. A non-targeting gRNA served as negative control (CTL). a: *AS1 (*p* = 0.0009), *EPHA2 (*p* = 0.0335), *ADM (*p* = 0.0359); b: *AS1 (*p* = 0.0012), *EPHA2 (*p* = 0.008), *ADM (*p* = 0.0428). **c** Western blot with (AS1)/without (-, CTL) LNA knockdown of *HIF1α-AS1* in two independent experiments using two different batches of HUVEC. GAPDH served as control. **d**–**g** Representative images (**d**, **f**) of a spheroid assay and quantification (**e**, **g**) of the cumulative sprout length of HUVECs treated with/without siRNAs against *EPHA2* (**d** and **e**) or LNA GapmeRs targeting *HIF1α-AS1* (**f** and **g**). Scale bar, 200 µm. One-Way ANOVA with Bonferroni test. e: CTL-VEGF-A (*n* = 12), CTL+VEGF-A (*n* = 15), EPHA2-VEGF-A (*n* = 13), EPHA2+VEGF-A (*n* = 12); *CTL-/+VEGF-A (*p* = <0.0001), *EPHA2-/+VEGF-A (*p* = <0.0001), *CTL/EPHA2+VEGF-A (*p* = <0.0001), *CTL/EPHA2-VEGF-A (*p* = 0.0079); g: CTL-basal (*n* = 21), AS1-basal (*n* = 26), CTL+VEGF-A (*n* = 19), AS1+VEGF-A (*n* = 23), CTL+bFGF (*n* = 12), AS1+bFGF (*n* = 32); *VEGF-A (*p* = 0.0495), *bFGF (*p* = 0.0012). **h**–**k** Quantification of the cumulative sprout length from the spheroid assays with siRNA targeting the *HIF1α-AS1* intron (**h**, *n* = 14 replicates), with CRISPRi (**i**, CTL-basal(*n* = 10), AS1-basal (*n* = 11), CTL+VEGF-A (*n* = 11), AS1+VEGF-A (*n* = 14), CTL+bFGF (*n* = 10), AS1+bFGF (*n* = 13)), with CRISPRa (**j**, CTL-basal (*n* = 8), AS1-basal (*n* = 7), CTL+VEGF-A (*n* = 10), AS1+VEGF-A (*n* = 7), CTL+bFGF (*n* = 5), AS1+bFGF (*n* = 10)) or after overexpression (**k**, CTL-basal (*n* = 21), 1200-basal (*n* = 22), CTL+VEGF-A (*n* = 20), 1200+VEGF-A (*n* = 18), CTL+bFGF (*n* = 21), 1200+bFGF (*n* = 19)) of the first 1200 nt of the *HIF1α-AS1* gene (included TFR2, named as 1200). One-Way ANOVA with Bonferroni test. h: *VEGF-A (*p* = 0.0109); i: *VEGF-A (*p* = <0.0001), *bFGF (*p* = 0.0006); j: *VEGF-A(*p* = 0.0429), *bFGF(*p* = 0.0489); k: *VEGF-A(*p* = <0.0001), *bFGF(*p* = 0.0004). **l**, **m** Western blot with (si) or without (CTL) siRNA-mediated knockdown of *HIF1α-AS1* targeting the intron (**l**) or with (OE) or without (CTL) overexpression of the first 1200 nt of *HIF1α-AS1* (**m**) in three independent experiments using three different batches of HUVEC. NONO served as control. **n**, **o**, **p** RT-qPCR of *TGFBR1* (**n**), *EPHA2* (**o**) or *ADM* (**p**) after replacement of *HIF1α-AS1*-TFR2 with single-stranded oligodeoxynucleotides (ssODN) containing *MEG3*-TFR or a DNA fragment of a luciferase negative control (Luc). -, no ssODN. *n* = 5 independent experiments, Paired *t* test. n: *(*p* = 0.0018); o: *MEG3(*p* = 0.0187), *Luc (*p* = 0.015); p: *(*p* = 0.0106). **q**, **r** ChIP with anti-S9.6 after replacement of *HIF1α-AS1*-TFR2 and qPCR for *EPHA2* (**q**) or *ADM* (**r**) TTS. -, no ssODN. One-Way ANOVA with Bonferroni test. *n* = 3 independent experiments. q: *MEG3 (*p* = 0.0025), *Luc (*p* =0 .0024); r: *Meg3 (*p* = 0.0006), *Luc (*p* = 0.0006). Data are presented as mean values ± SEM. AS1, HIF1α-AS1. M, marker. Source data (for c, l, m) are provided as a Source Data file.

The current data indicate that *HIF1α-AS1* forms triplexes with *EPHA2* and *ADM*, however, the mechanistic and functional consequences of this phenomenon are unclear. To investigate these aspects, gain and loss of function approaches were performed. Increasing the expression of *HIF1α-AS1* using a dCas9-VP64 CRISPR activation system (CRISPRa) reduced the expression of *EPHA2* and *ADM* (Fig. 4a). Conversely, downregulation of *HIF1α-AS1* with a dCas9-KRAB repression system (CRISPRi) increased the expression of *EPHA2* and *ADM* (Fig. 4b). Consistent with *HIF1α-AS1* repressing *EPHA2* and *ADM* gene expression, EPHA2 levels increased after knockdown of *HIF1α-AS1* (Figs. 2g, 4c). EPHA2 has a multi-faceted role in angiogenesis^29,33,34^. In HUVEC, knockdown of *EPHA2* with siRNAs strongly reduced its RNA and protein expression and inhibited angiogenic sprouting (Fig. 4d and e, Supplementary Fig. 4a–c). Conversely, a knockdown of *HIF1α-AS1* with LNA-GapmeRs increased VEGF-A- and bFGF-mediated angiogenic sprouting (Fig. 4f, g, Supplementary Fig. 4d), confirming the repressive effect of *HIF1α-AS1* on *EPHA2*. Additionally, CRISPRi targeting *HIF1α-AS1* or an siRNA-mediated knockdown of the *HIF1α-AS1* pre-RNA targeting the intron region next to the TFR2 were performed. Targeting the intron of *HIF1α-AS1* not only decreased the expression of TFR2, but also increased *EPHA2* and *ADM*, whereas *HIF1α* was not significantly altered (Supplementary Fig. 4e). As expected, both CRISPRi and siRNA against *HIF1α-AS1* intron induced VEGF-A-mediated sprouting (Fig. 4h and i, Supplementary Fig. 4f, g), whereas CRISPRa and an overexpression of the first 1200 nt of the *HIF1α-AS1* gene (containing Exon1, the beginning of the intron including TFR2) had the opposite effect (Fig. 4j and k, Supplementary Fig. 4h, i). The repressive effect of *HIF1α-AS1* on *EPHA2* was further confirmed by Western analysis, where siRNA-mediated knockdown of the *HIF1α-AS1* pre-RNA increased EPHA2 and overexpression of the first 1200 nt of the *HIF1α-AS1* gene decreased EPHA2 protein levels (Fig. 4l and m). The beneficial effect on sprouting is at least partially based on an anti-apoptotic effect as knockdown of *HIF1α-AS1* increased caspase 3&7 activity as measured by a cell-permeant fluorescent probe (SR-DEVD-FMK) that bound to active caspase 3 & 7 (Supplementary Fig. 4j). To demonstrate directly that TFR2 is responsible for the regulation of *EPHA2*, we replaced TFR2 by genome editing using a recombinant Cas9-eGFP, a gRNA targeting TFR2 and different single-stranded oligodeoxynucleotides (ssODN) harboring either the published *MEG3* TFR^4^ or a luciferase control sequence (Supplementary Fig. 4k). Replacement of the TFR2 with the *MEG3* TFR, which served as a positive control for a functional TFR repressing *TGFBR1* expression^4^, yielded a reduction in *TGFBR1* levels compared to the luciferase control (Fig. 4n). More importantly, the loss of TFR2 consequently led to a loss of *HIF1α-AS1* TFR2, an upregulation of *EPHA2* and partially of *ADM* (Fig. 4o, p, Supplementary Fig. 4l), and also ChIP with anti-S9.6 led to a reduced detection of the TTS of *EPHA2* and *ADM* (Fig. 4q, r). These data demonstrate that TFR2 is functional as a TFR and represses *EPHA2* and *ADM* gene expression.

### HIF1α-AS1 binds to and recruits HUSH to triplex targets

**Fig. 5:**
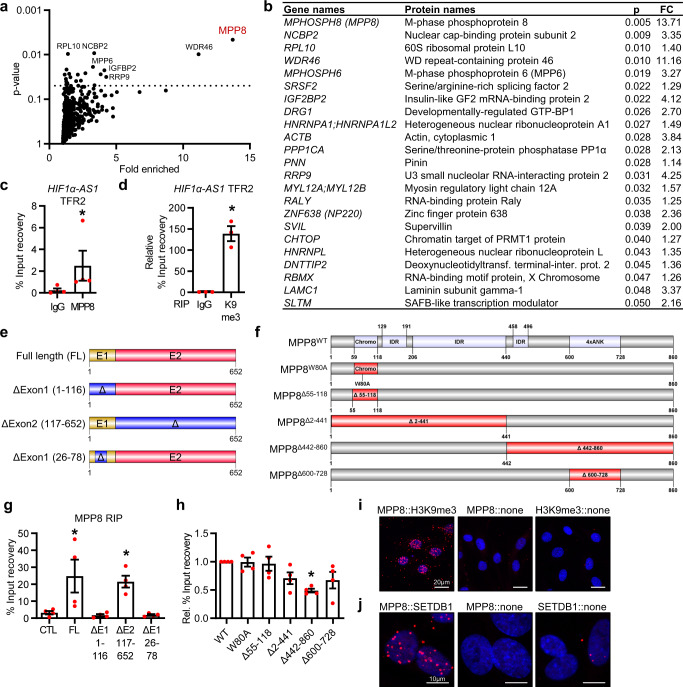
*HIF1α-AS1* interacts directly with the HUSH complex member MPP8. **a** Volcano plot of *HIF1α-AS1* protein interaction partners after RNA pulldown assay and ESI-MS/MS measurements with fold enrichment and *p* value. *n* = 5. Significant proteins are shown above the line (*p* < 0.05). **b** List of proteins enriched after RNA pulldown assay, their *p* value (p, unpaired *t* test, two-tailed) and absolute fold change (FC). **c** RIP with MPP8 antibodies and qPCR for *HIF1α-AS1* TFR2. IgG served as negative control. *n* = 4 independent experiments, Mann–Whitney test. *(*p* = 0.0286). **d** RIP with histone3-lysine9-trimethylation antibodies and qPCR for *HIF1α-AS1* TFR2. IgG served as negative control. *n* = 3 independent experiments, Paired *t* test. *(*p* = 0.0162). **e** Scheme of the different *HIF1α-AS1* RNAs used for in vitro RNA immunoprecipitation. E1, Exon1; E2, Exon2. **f** Scheme of the different MPP8 mutants used for in vitro RNA-immunoprecipitation. IDR intrinsically disordered region, ANK Ankyrin repeat; Chromo, Chromodomain. **g** RT-qPCR after in vitro binding assay of purified MPP8 with in vitro transcribed *HIF1α-AS1* RNAs. MPP8 antibodies were used for RNA immunoprecipitation (RIP). An T7-MCS in vitro transcribed RNA served as negative control (CTL). FL, full length; E1, Exon1; E2, Exon2. Δ indicates the deleted nt from *HIF1α-AS1* full length. *n* = 4 independent experiments, One-Way ANOVA with Dunnett’s test. *FL (*p* = 0.018), *ΔE2 117-652 (*p* = 0.0453). **h** RT-qPCR after in vitro binding assay of different in vitro translated His-tagged MPP8 mutants with in vitro transcribed *HIF1α-AS1* full length. Anti-His was used for RNA immunoprecipitation. *n* = 4 independent experiments, One-Way ANOVA with Dunnett’s test. *(*p* = 0.0029). **i**, **j** Proximity ligation assay of HUVECs with antibodies against MPP8 and H3K9me3 (**i**) or MPP8 and SETDB1 (**j**). The individual antibody alone served as negative control. Red dots indicate polymerase amplified interaction signals. Scale bar indicates 20 µm (**i**) or 10 µm (**j**). Images were representative of three independent experiments. Data are presented as mean values ± SEM.

To elucidate the mechanism by which *HIF1α-AS1* represses gene expression, *HIF1α-AS1*-associated proteins were studied using RNA pulldown experiments. 3’-biotinylated spliced *HIF1α-AS1* lncRNA or 3ʹ-biotinylated pcDNA3.1+ negative control were incubated in nuclear extracts from HUVECs and RNA-associated proteins were identified by electrospray ionization mass spectrometry, which retrieved M-phase phosphoprotein 8 (MPP8)-a component of the human silencing hub (HUSH) complex^35^- as top hit (Fig. 5a–b, Supplementary Data 6). The HUSH-complex is a nuclear machinery consisting of the chromodomain-containing protein MPP8, TASOR (FAM208A) and PPHLN1 (Periphilin), and was originally thought to mediate gene silencing during viral infection by recruiting the SET Domain Bifurcated Histone Lysine Methyltransferase 1 (SETDB1) which methylates H3K9^35^. The HUSH complex has not yet been studied in vascular cells and an interaction of its core protein MPP8 with lncRNAs has not been reported. To support our finding, RIP revealed that *HIF1α-AS1* and its TFR2, but not *HIF1A* mRNA, interact with MPP8 (Fig. 5c, Supplementary Fig. 5a–b). Furthermore, *HIF1α-AS1* was highly enriched with H3K9me3 (Fig. 5d).

To map the RNA binding region of MPP8 on *HIF1α-AS1*, we used *cat*RAPID fragments^36^, an algorithm involving division of polypeptide and nucleotide sequences into fragments to estimate the interaction propensity of protein-RNA pairs. This highlighted potential binding regions within Exon1 (Supplementary Fig. 5c). To substantiate these data experimentally, ex vivo bindings assays were performed between fragments of *HIF1α-AS1* and recombinant MPP8 (Fig. 5e) as well as with *HIF1α-AS1* and in vitro translated MPP8 mutants, among them the mutation in the chromodomain W80A, a chromodomain deletion, an N- or C-terminal half deletion and a deletion of the Ankyrin repeats (ANK) (Fig. 5f). MPP8 interacted directly with *HIF1α-AS1* full length and a *HIF1α-AS1* mutant lacking Exon2 (Fig. 5g). In contrast and in accordance with the *cat*RAPID prediction, deletion of Exon1 (nucleotides 26-78 in particular) prevented the interaction (Fig. 5g), indicating that this region of *HIF1α-AS1* is critical for the interaction of *HIF1α-AS1* with MPP8. On the protein side, RIP of the MPP8 mutants with anti-His antibodies followed by RT-qPCR for *HIF1α-AS1* revealed that the interaction of *HIF1α-AS1* with MPP8 was strongly reduced by a deletion of the C-terminal half of MPP8, but not by deletion or mutation of the chromodomain (Fig. 5h). Further, the Ankyrin repeats in the C-terminus seem to effect the interaction only to a minor extent (Fig. 5h). Uniprot^37^ listed three disordered regions, two of them in the N-terminal half and one in the C-terminal half (Fig. 5f), which could potentially be involved in the interaction.

To demonstrate that *HIF1α-AS1* acts through HUSH complex recruitment, we first tested whether parts of this complex exist in endothelial cells. Proximity ligation assays with antibodies against MPP8, dsDNA, H3K9me3 and SETDB1 confirmed the association of MPP8 with dsDNA (Supplementary Fig. 5d), H3K9me3 (Fig. 5i) and SETDB1 (Fig. 5j) in the nuclei of endothelial cells, indicating that parts of the complex are present at endothelial chromatin.

**Fig. 6:**
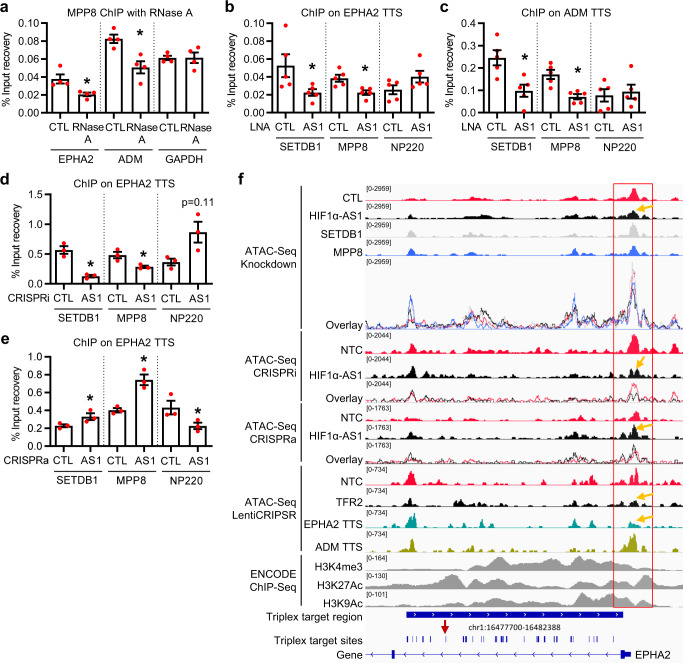
*HIF1α-AS1* directs the HUSH complex member MPP8 and SETDB1 to triplex target sites. **a** Chromatin immunoprecipitation (ChIP) with MPP8 antibodies with or without RNase A treatment and qPCR for the triplex target sites of *EPHA2* and *ADM*. Primers against a promoter sequence of GAPDH served as negative control. *n* = 4 independent experiments, paired *t* test. *EPHA2 (*p* = 0.0223), *ADM (*p* = 0.0221). **b**, **c** ChIP with antibodies against SETDB1, MPP8 or NP220 in HUVECs treated with (AS1) or without (CTL) LNA GapmeRs against *HIF1α-AS1*. QPCR was performed for *EPHA2* TTS (b) or *ADM* TTS (c). *n* = 5 independent experiments, paired *t* test. b: *SETDB1 (*p* = 0.0487), *MPP8 (*p* = 0.0287); c: *SETDB1 (*p* = 0.0358), *MPP8 (*p* = 0.0109). **d**, **e** ChIP with SETDB1, MPP8 or NP220 antibodies after CRISPRi (d) or CRISPRa (e) for *HIF1α-AS1* and qPCR for *EPHA2* TTS. *n* = 3 independent experiments, paired *t* test. d: *SETDB1 (*p* = 0.0371), *MPP8 (*p* = 0.0117); e: *SETDB1 (*p* = 0.0159), *MPP8 (*p* = 0.0465), *NP220 (*p* = 0.0202). **f** Genome tracks for *EPHA2* of ATAC-Seq in HUVECs separately and as an overlay after knockdown of *HIF1α-AS1* (black), SETDB1 (gray), MPP8 (blue) or the negative control (red), after CRISPRi and CRISPRa of *HIF1α-AS1* or after LentiCRISPR-mediated deletions of *HIF1α-AS1* TFR2, *EPHA2* TTS or *ADM* TTS. ChIP-Seq data (H3K4me3, H3K27Ac, H3K9Ac) in HUVECs was derived from ENCODE. Numbers in square brackets indicate data range values. Red arrow indicates the TTS analyzed in this study, orange arrows indicate strong changes. The red box indicates the relevant location. NTC non-targeting control, TFR triplex forming region, TTS triplex target site. Data are presented as mean values ± SEM.

ChIP with and without RNase A revealed that targeting of MPP8 and SETDB1, but not NP220, which is another protein associated with the HUSH complex^38^ and interacting with *HIF1α-AS1* (Fig. 5b), to the *HIF1α-AS1* TTS of *EPHA2* and *ADM* were attenuated after RNA depletion (Fig. 6a, Supplementary Fig. 6a, b). A region 5.7 kb downstream of *EPHA2*, which harbors different triplex target sites to the one studied here (Fig. 2h), also appeared to be reduced after RNase treatment the binding of MPP8 and SETDB1, but not NP220, indicating that MPP8 and SETDB1 might also act there (Supplementary Fig. 6c–e). To demonstrate the dependence of the interactions with the TTS on *HIF1α-AS1*, ChIP experiments with antibodies targeting SETDB1, MPP8 and NP220 after LNA-GapmeR-mediated knockdown, CRISPRi and CRISPRa of *HIF1α-AS1* were performed. The binding of SETDB1 and MPP8, but not of NP220, to the triplex target sites of *HIF1α-AS1 -*and not to the regions up- or downstream of the TTS- required the presence of the lncRNA (Fig. 6b-e, Supplementary Figs. 6f–i and 7a–j) suggesting that these interactions facilitate epigenetic processes and ultimately regulate gene expression. ATAC-Seq confirmed that these factors act in the region of the TTS: After knockdown of *HIF1α-AS1*, SETDB1 or MPP8, the chromatin accessibility of both the *EPHA2* and *ADM* transcriptional start sites were reduced, which was also seen by CRISPRi of *HIF1α-AS1* and by LentiCRISPR-mediated deletion of *HIF1α-AS1* TFR2 (Fig. 6f, Supplementary Figs. 7k and 8). LentiCRISPR-mediated deletion of *EPHA2* TTS or *ADM* TTS showed similar effects for the TSS of their gene locus and CRISPRa of *HIF1α-AS1* was confirmative by showing the opposite effect with increased open chromatin at the TSS of *EPHA2* and *ADM* (Fig. 6f, Supplementary Fig. 8). An increase in accessibility to the region downstream of the EPHA2 TTS was detected after knockdown of *HIF1α-AS1*, SETDB1 and MPP8; however, this could not be validated with CRISPRi/a for *HIF1α-AS1* or LentiCRISPR-dependent *HIF1α-AS1* TFR2, EPHA2 TTS or ADM TTS experiments, suggesting that the regions within the TSS, but not downstream of the EPHA2 TTS, may contain the most essential repressor regions. These data indicate that triplex formation by *HIF1α-AS1* is important for fine-tuning chromatin accessibility locally and thereby gene expression of *EPHA2* and *ADM* through SETDB1 and MPP8.

## Discussion

The present study combined molecular biology, bioinformatics, physiology and structural analysis to identify and establish the lncRNA *HIF1α-AS1* as a triplex-forming lncRNA in human endothelial cells. Through *trans*-acting triplex formation by a specific region within *HIF1α-AS1*, *EPHA2* and *ADM* DNA target sites are primed for their interaction with the HUSH complex members MPP8 and SETDB1 to mediate gene repression through control of chromatin accessibility. Physiologically, the anti-angiogenic lncRNA *HIF1α-AS1* is dysregulated in hypoxia and severe angiogenic and pulmonary diseases like CTEPH, IPAH and GBM. Thus, the present work establishes a putative link of a disease-relevant lncRNA and the HUSH complex by triplex formation resulting in the inhibition of endothelial gene expression.

The interaction of chromatin modifying complexes with lncRNAs suggests that lncRNAs have targeting or scaffolding functions within these complexes to modulate chromatin structure and thereby gene expression. Most studied lncRNAs have been identified to interact with complexes such as PRC2, SWI/SNF, E2F1 and p300, e.g. *MEG3*^4^*, FENDRR*^12^*, MANTIS*^39^, and *KHPS1*^7,10^. In the present work, we identified another silencing complex that can be targeted by lncRNAs: We demonstrated that *HIF1α-AS1* interacts with proteins of the HUSH complex, which mediates gene silencing. HUSH is also involved in silencing extrachromosomal retroviral DNA^38^. Recently it has been shown that the HUSH complex, particularly MPP8, which is downregulated in many cancer types and whose depletion caused overexpression of long interspersed element-1 (LINE-1s) and Long Terminal Repeats, controls type I Interferon signaling involving a mechanism with dsRNA sensing by MDA5 and RIG-I^40^. Here we report a direct interaction of the HUSH complex member MPP8 with *HIF1α-AS1*. Moreover, we identified Exon1 of *HIF1α-AS1* as being critical for this function. The HUSH complex has not yet been studied in vascular cells; it is not known whether its published composition with MPP8, TASOR and PPHLN1^35^ is valid for endothelial cells. Our data propose that, in endothelial cells, the HUSH complex member MPP8 interacts with H3K9me3 and DNA and that SETDB1 and MPP8, but not NP220, repress gene expression of *HIF1α-AS1*-specific target genes.

The finding that *HIF1α-AS1* interacts with the C-terminal domain of MPP8, and not with the chromodomain, was unexpected. The N-terminus of MPP8 was reported to interact with H3K9me3^41^. Substitution of Trp80 to alanine (W80A) within the chromodomain showed that it is important for H3K9me3 binding^41^, but also for other interactions, such as with DNMT3A^42^. Douse et al. removed the first 499 aa of MPP8 without impairing HUSH function; the function of HUSH was, however, only affected by the deletion of the amino acids 500-860, which also contain the predicted ankyrin repeats^43^. A similar finding was made for the maintenance of the self-renewal of ground-state murine embryonic stem cells, where not the chromodomain, but a C-terminal region of MPP8 was required for function^44^. The function of the IDRs within MPP8 were not investigated so far, but disordered regions are discussed to be potential linkers or binding partners of RNA^45^.

We propose that *HIF1α-AS1* mediates the anti-angiogenic effects through triplex-formation with the receptor tyrosine kinase *EPHA2* and the preprohormone *ADM* genes. EPHA2 is a major regulator of angiogenic processes since EphA2-deficient mice displayed impaired angiogenesis in response to ephrin-A1 stimulation in vivo^46^. EphA2-deficient endothelial cells failed to undergo cell migration and vascular assembly in response to ephrin-A1 and only adenovirus-mediated transduction of *Epha2* restored the defect^46^. Additionally, ADM promotes arterio- and angiogenesis^32^. Both genes were upregulated after *HIF1α-AS1* knockdown, explaining why *HIF1α-AS1* knockdown increased sprouting. However, other *HIF1α-AS1* targets are likely to contribute to the phenotype, such as the proangiogenic genes *HIF1A*^47^, *THBS1*^48^, *EGR1*^49^ or *NR2F2*^50^.

Our data indicate that the TSS region of *EPHA2*, where changes in chromatin accessibility were found consistently with knockdown, CRISPRi/a and LentiCRISPR, may contain repressive elements required to control the transcription of *EPHA2*. Other regions, such as the *EPHA2* region downstream of the triplex target sites, showed sensitivity to *HIF1α-AS1*, MPP8 and SETDB1 knockdown, but could not be validated by CRISPRi/a or LentiCRISPR. This finding could indicate that this region is probably sensitive to different transfection methods (electroporation versus transfection reagents) or the use of small RNA molecules, which was not the case in CRISPR experiments. The relevance of that region for the control of *EPHA2* transcription needs to be further clarified with experiments involving region-specific mutations.

In our unbiased approach, a large number of DNA binding sites were identified for *HIF1α-AS1* with triplex domain finder analysis. The large number is not unusual as many of these binding sites overlap and are not identical. Also for other lncRNAs, such as *GATA6-AS*, *FENDRR*, *HOTAIR* and *PARTICLE*, many DNA binding sites have been predicted within their target genes^9,14^. *EPHA2* and *ADM*, as well as *PLEC*, *RP11-276H7.2*, *MIDN* and *EGR1* contained a large number of DNA binding sites for *HIF1α-AS1* and were upregulated after *HIF1α-AS1* knockdown. It is therefore tempting to speculate that similar regulatory mechanisms may play a role in the regulation of these genes. Despite containing triplex target sites, several genes were unaffected by changing the expression level of the triplex-forming RNA. Given the large number of target sites, this could be a consequence of redundancy with respect to target sites or lncRNAs, steric hindering or additional local factors so far unknown. In fact, beyond Hoogsteen base pairing, the local factors required for triplex formation have not yet been identified. For example, it is possible that large protein complexes, like those involved in splicing interfere with binding. Also, the binding of transcription factors could compete with the lncRNA binding. Obviously, also the local epigenetic landscape and chromatin state impacts on triplex binding.

On several occasions, our study takes advantage on the fact that RNase H cleaves the RNA in DNA-RNA heteroduplexes^19^, and therefore enriches triplex-forming RNAs within the pool of DNA-interacting RNAs. Although this approach has been widely used in the field^4,6,7,13,15,20,51^, it is indirect and therefore not perfect. Proteins or specific local factors may shield RNAs resulting in false positive results and dynamic triplexes, with weak RNA interactions might also be digested. This is why additional methods, in particular bioinformatics prediction of Hoogsteen base pairing and ex vivo demonstration of triplex forming potential are needed to confirm the data obtained with the aid of differential RNase H-digestion.

The evidence for triplex formation by *HIF1α-AS1* is substantiated by a number of findings: Firstly, target recognition by *HIF1α-AS1* occurs via triplex formation involving GA-rich sequences of the DNA targets and GA-rich sequences within *HIF1α-AS1* lncRNA. This has also been observed for other lncRNAs such as *HOTAIR*^52^ and *MEG3*^4^. Secondly, the ^1^H-1D NMR and CD spectroscopy data for *HIF1α-AS1* provided similar but more detailed characteristics for triplex formation, compared with other studies^4,5^. Thorough NMR analysis of attenuations of the individual DNA Watson-Crick base-paired nucleotides allows delineation of those base pairs that are markedly affected by triplex formation. From a total of 25 base pairs in the *EPHA2*_DNA duplex target, only 13 base-pairs are affected. This observation in turn implies that not the entire *HIF1α-AS1* (TFO2-23) RNA is engaged in interaction with the DNA target duplex within the major groove of the DNA duplex, but substantial parts of the RNA strand retain dynamic flexibility which was further assured by our structural modeling. Through the use of heteroduplex samples, measurements at different temperatures, a reduction of equivalents of RNA and triplex analysis with stabilized DNA hairpin sequences, our study allowed an improved and extended analysis of triplex formation. Thirdly, in agreement with previous work^5^, most of the triplex target sites were located in the promoter region or introns of the DNA target genes. Fourthly, the triplex formation of *HIF1α-AS1* resulted in gene repression, a finding also observed for other triplex forming RNAs^3^. We could extend this finding by replacing the TFR2 of *HIF1α-AS1* with other sequences, which abolished the repressive effects.

*HIF1α-AS1* was downregulated in the lungs of patients with specific forms of pulmonary arterial hypertension (PAH). PAH is characterized by several structural changes, remodeling and lesion development in the pulmonary arteries. A study by Masri *et al*. demonstrated the impairment of pulmonary artery endothelial cells from IPAH patients to form tube-like structures^53^. CTEPH, a complex disorder with major vessel remodeling and small vessel arteriopathy, is characterized by medial hypertrophy, microthrombi formation and plexiform lesions^54^. It has been further shown that TGF-ß-induced angiogenesis was increased by circulating CTEPH microparticles co-cultured with pulmonary endothelial cells, indicating a pro-angiogenic feedback of endothelial injury^55^. Since *HIF1α-AS1* knockdown led to an increase in sprouting, we assume that the loss of *HIF1α-AS1* is a compensatory mechanism, which could be putatively included in the above mentioned pro-angiogenic feedback loop. *HIF1α-AS1* was also reduced in endothelial cells isolated from glioblastoma. Typically this pathology represents a highly angiogenic situation with defective endothelium and abnormal morphology^56^. Additionally, *HIF1α-AS1* is pro-apoptotic^27^ and so the reduction of *HIF1α-AS1* could explain the observed sprouting phenotype by the inhibition of apoptosis. Therefore, it is tempting to speculate that *HIF1α-AS1* harbors atheroprotective roles, which could be exploited to alter angiogenesis in patients. Strategies to design such therapeutics require data in other species and in different tissues. *HIF1α-AS1* is not endothelial-specific according to CAGE analysis. A comprehensive analysis on *HIF1α-AS1* conservation, especially of TFR2, is lacking. Initial attempts with BLAT showed that the first 1000 nt of the pre-processed *HIF1α-AS1* including TFR2 were conserved in primates and pigs, but not in rodents (data not shown). A potential application could be the promotion of vascular regeneration after an ischemia damage to promote early blood supply. Indeed, the post-ischemic healing response is not solely dependent on cardiomyocyte loss and adaptation but also on the damage response of the stroma-vascular compartment^57^. *HIF1α-AS1* was downregulated in hypoxia, but upregulated in the damage-relevant re-oxygenation phase. This suggests that specifically in that phase where endothelial proliferation is most needed, *HIF1α-AS1* limits the angiogenic response and therefore advocates itself as a target. Therefore, we propose an anti-HIF1α-AS1 approach to promote the early angiogenic response to promote post-ischemia regeneration.

Additionally, the data indicate that triplex formation could have therapeutic potential. The single nucleotide polymorphism (SNP) rs5002 (chr11:10326521 (hg19)) was found within the triplex target site of *ADM* with phenoscanner, which lists an association with hemoglobin concentration, red blood cell count and hematocrit^58^. Another link between a triplex forming lncRNA and PAH was reported by a massive upregulation of *MEG3* in paSMCs from IPAH patients. This prevented hyperproliferation after *MEG3* knockdown and a reduced apoptosis phenotype of IPAH-paSMCs involving a mechanism with miR-328-3p and IGF1R^59^. Although triplex formation was not studied, another study provided evidence that a ribonucleotide sequence can be used to form a potential triple helix to inhibit gene expression of the *IGF1R* gene in rat glioblastoma cells^60^. *MEG3* is known to impair cell proliferation and to promote apoptosis in glioma cells^61^. This argues that the binding of a lncRNA to DNA is potentially involved in PAH and GBM.

Taken together, the findings presented here highlight an important pathway of a scaffolding lncRNA within an epigenetic-silencer complex that has a crucial role in the regulation of endothelial genes.

## Methods

### Materials

The following chemicals and concentrations were used: Human recombinant VEGF-A 165 (R&D, 293-VE), Recombinant Human FGF-basic (154 a.a.) (bFGF, Peprotech, 100-18B), RNase A (NEB, EN0531), RNase H (NEB, M0297L), Cycloheximide (Sigma, C1988) and human recombinant TNF-α (Peprotech, 300-01 A). The following antibodies were used: Anti-H3-pan (Diagenode, C15200011), Anti-dsDNA [35I9 DNA] (Abcam, ab27156), Anti-DNA-RNA Hybrid [S9.6] (Kerafast, ENH001), Anti-EPHA2 (Bethyl, A302-025-M), Anti-GAPDH (Sigma, G8795), Anti-HSC70/HSP70 (Enzo Life Sciences, ADI-SPA-820), Anti-NONO (Bethyl, A300-587A), Anti-MPP8 (Bethyl, A303-051A-M), Recombinant Anti-6X His tag® antibody [EPR20547] (Abcam, ab213204, ChIP grade), Anti-H3K9me3 (Diagenode, SN-146-100), Anti-SETDB1 (Bethyl, A300-121A, for chromatin immunoprecipitation; Santa Cruz Biotechnology, ESET (G-4): sc-271488, for Proximity ligation assay) and Anti-ZNF638/NP220 (Bethyl, A301-548A-M).

### Cell culture

Pooled human umbilical vein endothelial cells (HUVECs) were purchased from PromoCell (C-12203, Lot No. 405Z013, 408Z014, 416Z042, Heidelberg, Germany) and originate from umbilical cord/ umbilical vein of caucasians (405Z013: 2 males, 1 female; 408Z014: 2 males, 1 female; 416Z042: 2 males, 2 females). HUVECs were cultured in a humidified atmosphere of 5% CO_2_ at 37 °C. Fibronectin-coated (356009, Corning Incorporated, USA) dishes were used to culture the cells. Endothelial growth medium (EGM), consisting of endothelial basal medium (EBM) supplemented with human recombinant epidermal growth factor (EGF), EndoCGS-Heparin (PeloBiotech, Germany), 8% fetal calf serum (FCS) (S0113, Biochrom, Germany), penicillin (50 U/mL) and streptomycin (50 µg/mL) (15140-122, Gibco/ Lifetechnologies, USA) was used. For each experiment except ATAC-Seq, at least three different batches of HUVEC from passage 3 were used. In case of hypoxic treatments, cells were incubated in a SciTive Workstation (Baker Ruskinn, Leeds, UK) at 0.1% O_2_ and 5% CO_2_ for the times indicated.

Human embryonic kidney 293 cells (HEK293) (ATCC, Manassas, USA) and Lenti-X 293 T cells (Takara, 632180, Japan) were cultured in Dulbecco’s Modified Eagle Medium High Glucose (Gibco) supplemented with 8% FCS, penicillin (50 U/mL) and streptomycin (50 μg/mL) (15140‐122, Gibco/ Lifetechnologies, USA), in a humidified atmosphere of 5% CO_2_ at 37 °C.

### Analyses of Triplex-Seq data to identify candidate lncRNAs

Triplex-Seq data of U2OS and HeLa S3 was used from^15^, aligned using STAR^62^ and peak-calling was performed with MACS2^63^. Peaks were intersected with Ensembl hg38 gene coordinates to produce a list of gene-associated peaks, which was filtered for lncRNAs. The overlap of U2OS and HeLa S3 lncRNAs was filtered for high confidence candidates by applying two cut-off filters for fold enrichment (>10) and -log10(P value peak enrichment) (>20). The *P* value for the enrichment of the peak (i-log10(*P* value peak enrichment)) was calculated using a Poisson distribution to estimate the expected number of reads which should lie within the peak region. The enrichments (fold enrichment, P) are then calculated based on the ratio of observed reads at the peak location (i.e. the real peak) vs. the expected peak. Next, the candidates were filtered for the presence of a nuclear value (>0) in ENCODE and for the presence of a signal (>0) in aorta, artery, lymphatic, microvascular, thoracic, umbilical vein and vein in FANTOM5 CAGE data^21–23^. Subsequently, the remaining candidates (*RMRP*, *HIF1α-AS1*, *RP5-857K21.4*, *SCARNA2* and *SNHG8*) were tested for their non-coding probability with the online tools CPAT^64^ and CPC2^65^. Lastly, regions enriched in the Triplex-Seq were manually inspected in the IGV browser to rule out the possibility that the signals belong to overlapping genes.

### Total and nuclear RNA isolation, Reverse transcription and RT-qPCR

Total RNA isolation was performed with the RNA Mini Kit (Bio&Sell). Reverse transcription was performed with SuperScript III Reverse Transcriptase (Thermo Fisher) and oligo(dT)23 together with random hexamer primers (Sigma). CopyDNA amplification was measured with RT-qPCR using ITaq Universal SYBR Green Supermix and ROX as reference dye (Bio-Rad, 1725125) in an AriaMX cycler (Agilent). Relative expression of target genes was normalized to ß-*Actin* or *18* *S* ribosomal RNA. Expression levels were analyzed by the delta-delta Ct method with the Agilent Aria 1.7 qPCR software. Oligonucleotides used for amplification are listed in Table 1.

**Table 1:** List of primers for RT-qPCR

Name	Forward primer (5ʹ−3ʹ)	Reverse primer (5ʹ−3ʹ)
b-actin	AAAGACCTGTACGCCAACAC	GTCATACTCCTGCTTGCTGAT
HIF1α-AS1 (TFR2)	CCGAAATCCCTTCTCAGCAG	TCTGTGTTTAGCGGCGGAGG
HIF1α-AS1 (E1)	GCCCTCCATGGTGAATCGGTCCCCGCG	CCTTCTCTTCTCCGCGTGTGGAGGGAG
HIF1α-AS1 (E2)	AGGGCTGTTCCATGTTTAGG	GTCTATGGATGCCCACATGC
HIF1α-AS1 (E1-I)	GCCCTCCATGGTGAATCGGTCCCCGCG	CAACCGAAATCCCTTCTCAGCAGCG
RMRP	TCCGCCAAGAAGCGTATCCC	ACAGCCGCGCTGAGAATGAG
SCARNA2	AGTGTGAGTGGACGCGTGAG	AAGTGTAAGCGGGAGGAGGG
RP5-857K21.4	AGAGTGAGGAGAAGGCTTAC	TTCTGAGTCCCAGAGGTTAC
HIF1α	GCTCATCAGTTGCCACTTCC	ACCAGCATCCAGAAGTTTCC
18 S rRNA	CTTTGGTCGCTCGCTCCTC	CTGACCGGGTTGGTTTTGAT
HIF1α-AS1 (TFR1)	TCAGACGAGGCAGCACTGTGCACTGAGG	TCGCTCGCCATTGGATCTCGAGGAACCC
HIF1α-AS1 (TFR3)	GAGCCCTAATCATAGGACTG	AGGGTCTGAGGTTTGAGTTC
KLF10	AGCCAGCATCCTCAACTATC	GCAGCACTTGCTTTCTCATC
SPHK1	GGAGATGCGCTTCACTCTGG	GGAGGCAGGTGTCTTGGAAC
CSRNP1	TGTGGCTGTCACTGCGATAG	TGTGGTCCATCTGGCACTTG
INTS6	GCCTGGCACCATGTCAGTAG	GCACCAAGGACTCCAGACAC
GATA2	GCAACCCCTACTATGCCAACC	CAGTGGCGTCTTGGAGAAG
IER5	AGACCGGGAACGTGGCTAAC	TCTCAGCACCGGCTTATCGC
YWHAZ	GTGTTCTATTATGAGATTCTGAAC	ATGTCCACAATGTCAAGTTGTCTC
THBS1	TGTACGCCATCAGGGTAAAG	AAGAAGGTGCCACTGAAGTC
EGR1	ACCCAGCAGCCTTCGCTAAC	AGAAGCGGCGATCACAGGAC
MIDN	AAGACACCCGGCTCAGTTCG	TGAGACATGAGGCCCGCTTC
EPHA2	GGCTGAGCGTATCTTCATTG	ACTCGGCATAGTAGAGGTTG
RP11-276H7.2	CCAGACTCCCTTTGCCTACC	GCAGAGAAGACCCACGTACC
PLEC	CCAAGGGCATCTACCAATCC	CACTCCAGCCTCTCAAACTC
ADM	TTCCGTCGCCCTGATGTACC	ATCCGCAGTTCCCTCTTCCC
TGFBR1	GAGCGGTCTTGCCCATCTTC	TTCAGGGGCCATGTACCTTTT

For nuclear RNA isolation, cells were resuspended in buffer A1 (10 mM HEPES pH 7.6, 10 mM KCl, 0.1 mM EDTA pH 8.0, 0.1 mM EGTA pH 8.0, 1 mM DTT, 40 µg/mL PMSF) and incubated on ice for 15 min. Nonidet was added to a final concentration of 0.75% and cells were centrifuged (1 min, 4 °C, 16,000 × *g*). The pellet was washed twice in buffer A1, lysed in buffer C1 (20 mM HEPES pH 7.6, 400 mM NaCl, 1 mM EDTA pH 8.0, 1 mM EGTA pH 8.0, 1 mM DTT, 40 µg/mL PMSF) and centrifuged (5 min, 4 °C, 16,000 × *g*). The supernatant was used for RNA isolation with RNA Isolation the RNA Mini Kit (Bio&Sell).

### Knockdown procedures

For small interfering RNA (siRNA) treatments, endothelial cells (80–90% confluent) were transfected with GeneTrans II according to the instructions provided by MoBiTec (Göttingen, Germany). The following siRNAs were used: siEPHA2 (Thermo Fisher Scientific, HSS176396), siSETDB1 (Thermo Fisher Scientific, s19112) and siMPP8 (Thermo Fisher Scientific, HSS123184). The stealth siRNA targeting the intron of *HIF1α-AS1* (approx. 100 nt downstream of TFR2) was designed with the Invitrogen BLOCK-iT RNAi designer (Thermo Fisher) and had the following sequence: 5ʹ-GCC TGG TCC CAA ACA TGC ATC ATA T-3ʹ. As negative control, scrambled Stealth RNAi™ Med GC (Life technologies) was used. All siRNA experiments were performed for 48 h.

For Locked nucleic acid (LNA)-GapmeR (Exiqon) treatment, the transfection was performed with the Lipofectamine RNAiMAX (Invitrogen) transfection reagent according to manufacturer’s protocol. All LNA-GapmeR transfections were performed for 48 h. LNA-GapmeRs were designed with the Exiqon LNA probe designer and contained the following sequences: *HIF1α-AS1* (1) 5ʹ-GAAAGAGCAAGGAAC A-3ʹ and as a negative Control 5ʹ-AACACGTCTATACGC-3ʹ.

### Protein isolation and western blot analyses

HUVECs were washed in Hanks solution (Applichem) and afterwards lysed with Triton X-100 buffer (20 mM Tris/HCl pH 7.5, 150 mM NaCl, 10 mM NaPPi, 20 mM NaF, 1% Triton, 2 mM Orthovanadat (OV), 10 nM Okadaic Acid, protein-inhibitor mix (PIM), 40 µg/mL Phenylmethylsulfonylfluorid (PMSF)). The cells were centrifuged (10 min, 16,000 × *g*) and protein concentration of the supernatant was determined with the Bradford assay. The cell extract was boiled in Laemmli buffer and equal amounts of protein were separated with SDS-PAGE. The gels were blotted onto a nitrocellulose membrane and blocked in Rotiblock (Carl Roth, Germany). After incubation with the first antibody, infrared-fluorescent-dye-conjugated secondary antibodies (Licor, Bad Homburg, Germany) were used and signals detected with an infrared-based laser scanning detection system (Odyssey Classic, Licor, Bad Homburg, Germany). Images were acquired with Image Studio 5.2 (Licor). The following first antibodies and dilutions were used: Anti-EPHA2 (Bethyl, A302-025-M, 1:1000), Anti-GAPDH (Sigma, G8795, 1:10000), Anti-HSC70/HSP70 (Enzo Life Sciences, ADI-SPA-820, 1:2000) and Anti-NONO (Bethyl, A300-587A, 1:5000). The following secondary antibodies and dilutions were used: IRDye® 680RD Donkey anti-Rabbit IgG Secondary Antibody (LICOR, 926-68073, 1:15000), IRDye® 800CW Donkey anti-Rabbit IgG Secondary Antibody (LICOR, 926-32213, 1:15000), IRDye® 680RD Donkey anti-Mouse IgG Secondary Antibody (LICOR, 926-68072, 1:15000) and IRDye® 800CW Donkey anti-Mouse IgG Secondary Antibody (LICOR, 926-32212, 1:15000).

### Human lung samples

The study protocol for tissue donation from human idiopathic pulmonary hypertension patients was approved by the ethics committee (Ethik Kommission am Fachbereich Humanmedizin der Justus Liebig Universität Giessen) of the University Hospital Giessen (Giessen, Germany) in accordance with national law and with Good Clinical Practice/International Conference on Harmonisation guidelines. Written informed consent was obtained from each individual patient or the patient’s next of kin (AZ 31/93, 10/06, 58/15)^66^.

Human explanted lung tissues from subjects with IPAH, CTEPH or control donors were obtained during lung transplantation. Samples of donor lung tissue were taken from the lung that was not transplanted. All lungs were reviewed for pathology and the IPAH lungs were classified as grade III or IV.

### PASMC isolation and culture

Pulmonary arterial smooth muscle cells (PASMCs) were handled and treated as described before^67^. Briefly, segments of PASMCs, which were derived from human pulmonary arteries (<2 mm in diameter) of patients with IPAH or from control donors, were cut to expose them to the luminal surface. Gentle scraping with a scalpel blade was used to remove the endothelium. The media was peeled away from the underlying adventitial layer. 1–2 mm^2^ sections of medial explants were cultured in Promocell smooth Muscle Cell Growth Medium 2 (Promocell, Heidelberg, Germany). For each experiment, cells from passage 4-6 were used. A primary culture of human PASMCs was obtained from Lonza (CC-2581, Basel, Switzerland), grown in SmGM-2 Bulletkit medium (Lonza) and cultured in a humidified atmosphere of 5% CO_2_ at 37 °C. Cells from passages 4–6 were used for experiments. For hypoxia experiments, PASMCs were incubated in hypoxia or normoxia chambers for 24 h in hypoxic medium (basal medium containing 1% FCS for human PASMCs). Hypoxia chambers were equilibrated with a water-saturated gas mixture of 1% O_2_, 5% CO_2_, and 94% N_2_ at 37 °C.

### Brain microvessel isolation from glioblastoma (GBM) patients

Studies for human glioblastoma were covered by an ethics statement according to the guidelines of the University of Frankfurt, whose approval number for autopsy material is GS-249/11 and for resection material GS-04/09. Human Brain microvessel (HMBV) isolation from GBM patients was performed exactly as described before^39^. Within 3 h post surgery, fresh brain specimens were obtained from GBM patients. For patients without available normal appearing healthy tissue, healthy material was obtained from epilepsy or dementia patients or autopsy material within a day postmortem. To isolate HMBV, specimens obtained in ice-cold MVB (15 mM HEPES, 147 mM NaCl, 4 mM KCl, 3 mM CaCl_2_, 1.2 mM MgCl_2_, 5 mM glucose and 0.5% BSA, pH 7.4) were used. These were cleared using forceps and the tissue was homogenized in 3-fold ice-cold MVB buffer by 15 up and down strokes in a tight-fitting douncer (0.25 mm clearance, 10 mL Wheaton) attached to an electrical overhead stirrer (2000 rpm, VOS 14, VWR). The homogenate was centrifuged (400 × *g*, 10 min, 4 °C) and the pellet was resuspended in fourfold 25% BSA (in PBS). After an additional centrifugation (2000 × *g*, 30 min, 4 °C), myelin fat in the top layer was aspirated. Next, the pellet containing the microvessels was resuspended in 3 mL ice-cold MVB/ gram starting material. To remove large vessels and tissue aggregates, the sample was filtered through 100-micron sterile nylon mesh cell strainer (BD) and the microvessels were trapped onto a 40-micron sterile nylon mesh (BD). Afterwards, the mesh was washed once with ice-cold MVB and the microvessels were lysed directly with ice-cold RLT-Plus RNA lysis buffer (Qiagen), vortexed and stored at −80 °C until use.

### CRISPR/dCas9 activation (CRISPRa) and inactivation (CRISPRi)

Guide RNAs (gRNA) were designed with the help of the web-interfaces of CRISPR design (http://crispr.mit.edu/). CRISPR activation (CRISPRa) was performed with a catalytically inactive Cas9 (dCas9), which is fused to the transcription activator VP64 (pHAGE EF1α dCas9-VP64), whereas CRISPRi was performed with a dCas9 fusion to the KRAB repressive domain. Both were used together with a sgRNA(MS2) vector containing the individual guide RNA (gRNA) to induce or repress *HIF1α-AS1* gene expression. pHAGE EF1α dCas9-VP64 and pHAGE EF1α dCas9-KRAB were a gift from Rene Maehr and Scot Wolfe (Addgene plasmid # 50918, # 50919)^68^ and sgRNA(MS2) cloning backbone was a gift from Feng Zhang (Addgene plasmid # 61424)^69^. The following oligonucleotides were used for cloning of the guide RNAs into the sgRNA(MS2) vector: For CRISPRa of *HIF1α-AS1* 5ʹ-CACCGGGGC CGGCCTCGGCGTTAAT-3ʹ and 5ʹ-AAACATTAACGCCGAGGCCGGCCCC-3ʹ, and for CRISPRi of *HIF1α-AS1* 5ʹ-CACCGGTCTGGTGAGGATCGCATGA-3ʹ and 5ʹ-AAACTCATGCGATCCTCACCAGACC-3ʹ. After cloning, plasmids were purified and sequenced. The transfection of the plasmids in HUVEC was performed using the NEON electroporation system (Invitrogen).

### CRISPR-Cas9 genome editing with LentiCRISPR

Guide RNAs (gRNA) were selected using the publicly available CRISPOR algorithm 5.01 (http://crispor.tefor.net/)^70^. A dual gRNA approach consisting of gRNA-A and gRNA-B was used to facilitate the individual deletions. The gRNAs were cloned into lentiCRISPRv2 vector backbone with Esp3I (Thermo Fisher, FD0454) according to the standard protocol^71^. lentiCRISPRv2 was a gift from Feng Zhang (Addgene plasmid #52961; http://n2t.net/addgene:52961; RRID:Addgene_52961)^71^.

For annealing, the following oligonucleotides were used: *HIF1α-AS1 TFR2*: gRNA-A, 5ʹ-CACCGGCTCGTCTGTGTTTAGCGG-3ʹ and 5ʹ-AAACCCGCTAAACACAGACGAGCC-3ʹ, gRNA-B, 5ʹ-CACCGGTGCGGCTCAGCCCGAGTC-3ʹ and 5ʹ-AAACGACTCGGGCTGAGCCGCACC-3ʹ; *EPHA2 TTS*: gRNA-A, 5ʹ-CACCGTTGCATAGGTTCTATGCCC-3ʹ and 5ʹ-AAACGGGCATAGAACCTATGCAAC-3ʹ, gRNA-B, 5ʹ- CACCGAAGTGCTACCCTCCCTAGA-3ʹ and 5ʹ-AAACTCTAGGGAGGGTAGCACTTC-3ʹ; *ADM TTS*: gRNA-A, 5ʹ- CACCGCCGAGAGCAGGAGCGCGCG-3ʹ and 5ʹ-AAACCGCGCGCTCCTGCTCTCGGC-3ʹ, gRNA-B, 5ʹ- CACCGCGCGTGGCTGAGGAAAGAA-3ʹ and 5ʹ-AAACTTCTTTCCTCAGCCACGCGC-3ʹ. After cloning, the gRNA-containing LentiCRISPRv2 vectors were sequenced and purified. Lentivirus was produced in Lenti-X 293 T cells (Takara, 632180) using Polyethylenamine (Sigma-Aldrich, 408727), psPAX2 and pVSVG (pMD2.G). pMD2.G was a gift from Didier Trono (Addgene plasmid #12259; http://n2t.net/addgene:12259; RRID:Addgene_12259). psPAX2 was a gift from Didier Trono (Addgene plasmid #12260; http://n2t.net/addgene:12260; RRID:Addgene_12260). LentiCRISPRv2-produced virus was transduced in HUVEC with polybrene transfection reagent (MerckMillipore, TR-1003-G) and selection was performed with puromycin (1 μg/mL) for 6 d. Afterwards, genomic DNA was isolated, PCR was performed followed by agarose gel electrophoresis and ethidiumbromide staining. The following primers were used: *HIF1α-AS1* TFR2 del, 5ʹ-GCGGAGGAAAGAGAAAGGAG-3ʹ and 5ʹ-GAACAGAGAGCCCAGCAGAG-3ʹ; EPHA2 TTS del, 5ʹ-TCTCCTTACCCTCTAGGGAG-3ʹ and 5ʹ-ATTCTAGGCCCAGAGACCAG-3ʹ; ADM TTS del, 5ʹ-GCGTGGCTGAGGAAAGAAAG-3ʹ and 5ʹ-GAGAGTGATCTGCCAAGTAC-3ʹ; GAPDH, 5ʹ-TGGTGTCAGGTTATGCTGGGCCAG-3ʹ and 5ʹ- GTGGGATGGGAGGGTGCTGAACAC-3ʹ.

### CRISPR-Cas9 ArciTect genome editing

For genome editing, the ArciTect Cas9-eGFP system was used according to the manufacturer’s conditions (STEMCELL Technologies, Köln, Germany). Briefly, ArciTect™ CRISPR-Cas9 RNP Complex solution was generated with 60 μM gRNA and tracrRNA and 3.6 µg ArciTect™ Cas9-eGFP Nuclease. Afterwards, 20 µM single-strand oligodeoxynucleotide (ssODN) was added to the RNP solution. The following gRNA was used to target TFR2 of *HIF1α-AS1*: 5ʹ-ACGTGCTCGTCTGTGTTTAG-3ʹ. The following ssODNs (Integrated DNA Technologies, Leuven, Belgium) were used to replace TFR2: MEG3, 5ʹ-GAG GCACAGCTGGGACGGGCTGCGACGCTCACGTGCTCGTCTGTGTTGTAATCGCTCCCTCTCTGCTCTCCGATGGGGGTGCGGCTCAGCCCGAGTCTGGGGACTCTGCGCCTTCTCCGAAGGAAGGCGG-3ʹ, negative control Luc 5ʹ-GCTGAGGCACAGCTGGGACGGGCTGCGACGCTCACGTGCTCGTCTGTGTTGTAATTATCACGCTCGTCGTTCGGTATGATGGGGGTGCGGCTCAGCCCGAGTCTGGGGACTCTGCGCCTTCTCCGAAGGAAG-3ʹ. 400.000 HUVECs were seeded in a 12-well plate and electroporated in E2 buffer with the NEON electroporation system (Invitrogen) (1,400 V, 1 × 30 ms pulse). A full medium exchange was done every 24 h and cells were incubated for 72 h. For FACS, eGFP-positive cells were sorted in PBS supplemented with 5% FCS with a Cell Sorter SH800S (Sony).

### HIF1α-AS1 mutants, pCMV6-MPP8-10xHis and MPP8 mutants

To clone pcDNA3.1 + HIF1α-AS1, *HIF1α-AS1* was amplified with PCR from cDNA (forward primer: 5ʹ-ATATTAGGTACCCGCCGCCGGCGCCCTCCATGGTG-3ʹ, reverse primer: 5ʹ-ACGGGAATTCTAATGGAACAT TTCTTCTCCCTAG-3ʹ) and insert and vector (pcDNA3.1+) were digested with Acc65I/EcoRI and ligated. pCMV6-MPP8-MYC-DDK was obtained from Origene (#RC202562L3). The plasmid pcDNA3.1 + HIF1α-AS1_1200 (called TFR2) included the first 1200 nt (hg19, chr14:62,161,342-62,162,541) of the genomic DNA of the *HIF1α-AS1* gene and was synthesized from Biomatik (Canada).

To create pcDNA3.1 + HIF1-AS1-Δexon1 (1-116), pcDNA3.1 + HIF1-AS1-Δexon2 (117-652), pcDNA3.1 + HIF1-AS1-Δexon1 (26-78) and pCMV6-MPP8-10xHIS (replacement of c-terminally MYC-DDK by 10xHIS), site-directed mutagenesis was performed with the Q5 Site-Directed Mutagenesis Kit (NEB) according to the instructions of the manufacturer. Oligonucleotides and annealing temperatures for mutagenesis were calculated with the NEBaseChanger online tool from NEB. The pcDNA3.1 + HIF1α-AS1 and pCMV6-MPP8-Myc-DDK plasmids served as templates and were amplified with PCR with the following oligonucleotides to obtain the individual constructs: for pcDNA3.1 + HIF1α-AS1-Δexon1 (1-116), 5ʹ-ACTACAGTTCAACTGTCAATTG-3ʹ and 5ʹ-GGTACCAAGCTTAAGTTTAAAC-3ʹ, for pcDNA3.1 + HIF1-AS1-Δexon2 (117-652), 5ʹ-GAATTCTGCAGATATCCAG-3ʹ and 5ʹ-CTTTCCTTCTCTTCTCCG-3ʹ, for pcDNA3.1 + HIF1α-AS1-Δexon1 (26-78), 5ʹ-AGCGCTGGCTCCCTCCAC-3ʹ and 5ʹ-TTCACCATGGAGGGCGCC-3ʹ, for pCMV6-MPP8-10xHIS, 5ʹ-CACCATCATCACCACCATCACTAAACGGCCGGCCGCGGTCAT-3ʹ and 5ʹ-GTGATGGTGAGAGCCTCCACCCCCCTGCAGCTGCACTCTGTATGCACCTATTAGC-3ʹ. The plasmids were verified by sequencing.

The individual MPP8 mutants were generated with the Q5 Site-Directed Mutagenesis Kit (#E0554S, NEB) according to the instructions of the manufacturer. To generate primer sequences and calculate annealing temperatures, the NEBaseChanger™ (NEB) was used. The pCMV6-MPP8-10xHis plasmid served as template and was amplified with PCR with the following oligonucleotides to obtain the individual mutants: W80A, 5ʹ-CAAAGTTCGCgcGAAAGGCTATAC-3ʹ and 5ʹ-TAAAGAACTTTACCCCCC-3ʹ, Δ55-118, 5ʹ-AGGAAGGATATTCAGAGACTATCC-3ʹ and 5ʹ-GTCCTCCTCACTGTCGCC-3ʹ, Δ2-441, 5ʹ-AAGGAAATCAGAAATGCATTTGATTTATTTAAATTAACTCCAGAAGAAAAAAATGATGTTTCTG-3ʹ and 5ʹ-CATGGCGATCGCGGCGGC-3ʹ, Δ442-860, 5ʹ-GGGGGTGGAGGCTCTCAC-3ʹ and 5ʹ-AAGTGTCTTTAATCCTTTTGGCTCTTTTCTG-3ʹ, Δ600-728, 5ʹ-GTAGCAGAAGAGACAATAAAG-3ʹ and 5ʹ-GGAATCCTCTTGGTCCAG-3ʹ. The final plasmids were verified by sequencing. In vitro protein synthesis of the MPP8 mutants was performed with the PURExpress kit (E6800, NEB) according to the manufacturer’s protocol.

### Purification of pCMV6-MPP8-10xHis

To generate purified MPP8-10xHIS protein, pCMV6-MPP8-10xHIS was overexpressed in HEK293 by transfection with Lipofectamine 2000 according to the manufacturer’s protocol. After 24 h, cells were lysed with three cycles of snap freezing in liquid nitrogen and 2% triton X-100 with protease inhibitors. Recombinant MPP8-10xHis was purified using HisTrap FF crude columns (Cytiva Europe, Freiburg, Germany, #11000458) with a linear gradient of imidazole (from 20 to 500 mM, Merck, Burlington, United States, #104716) in an Äkta Prime Plus FPLC system (GE Healthcare/Cytiva Europe).

### In vitro transcription and RNA 3ʹend biotinylation

Prior to in vitro transcription, pcDNA3.1 + HIF1α-AS1, pcDNA3.1 + HIF1α-AS1-Δexon1 (1-116), pcDNA3.1 + HIF1α-AS1-Δexon2 (117-652), pcDNA3.1 + HIF1α-AS1-Δexon1 (26-78) or control pcDNA3.1+ were linearized with SmaI (Thermo Fisher, FD0663). After precipitation and purification of linearized DNA, RNA was in vitro transcribed according to the manufacturers protocol with T7 Phage RNA Polymerase (NEB), and DNA was digested with RQ DNase I (Promega). The remaining RNA was purified with the RNeasy Mini Kit (Qiagen) and used for binding reactions with MPP8-10xHis in RIP experiments. For RNA pulldown experiments, RNA of *HIF1α-AS1* or of the control pcDNA3.1+ were further biotinylated at the 3ʹend with the Pierce RNA 3ʹend biotinylation kit (Thermo Fisher).

### RNA pulldown assay and mass spectrometry

The RNA pulldown assay was performed similar to^39^. For proper RNA secondary structure formation, 150 ng of 3ʹend biotinylated *HIF1α-AS1* or control RNA was heated for 2 min at 90 °C in RNA folding buffer (10 mM Tris pH 7.0, 0.1 M KCl, 10 mM MgCl_2_), and then put on RT for 20 min. 1 × 10^7^ HUVECs were used per sample. Isolation of nuclei was performed with the truCHIP™ Chromatin Shearing Kit (Covaris, USA) according to the manufacturers protocol without shearing the samples. Folded Bait RNA was incubated in nuclear cell extracts for 3 h at 4 °C. After incubation, samples were UV crosslinked. Afterwards, Streptavidin M-270 Dynabeads (80 µL Slurry, Thermo Fisher) were incubated with cell complexes for 2 h at 4 °C. After 4 washing steps with the lysis buffer of the truCHIP chromatin Shearing Kit (Covaris, USA), beads were put into a new Eppendorf tube. For RNA analysis, RNA was extracted with TRIzol (Thermo Fisher). Afterwards, RNA purification was performed with the RNeasy Mini Kit (Qiagen). If indicated, RT-qPCR was performed. For mass spectrometric measurements in order to reduce complexity, samples were eluted stepwise from the beads. Beads were resuspended in 50 mM ammoniumhydrogencarbonate and 1 µL RNAse A. Supernatant was reduced and alkylated with DTT and chloracetamid, respectively. Remaining Beads were resuspended in 20 µL 6 M Guanidinhydrochlorid (GdmCl), 100 mM Tris/HCl, pH 8.5, 10 mM DTT and incubated at 95 °C for 5 min. Reduced thiols were alkylated with 40 mM chloroacetamid and samples were diluted with 25 mM Tris/HCl, pH 8.5, 10% acetonitrile to obtain a final GdmCl concentration of 0.6 M. Proteins of both fractions were digested with 1 µg Trypsin/LysC (sequencing grade, Promega) overnight at 37 °C under gentle agitation. Digestion was stopped by adding trifluoroacetic acid to a final concentration of 0.1%. Peptides were loaded on multi-stop-and-go tip (StageTip) containing three a stack of three C18-disks. Both fractions were eluted in wells of microtiter plates and peptides were dried and resolved in 1% acetonitrile, 0.1% formic acid. Liquid chromatography/mass spectrometry (LC/MS) was performed on Thermo Scientific™ Q Exactive Plus equipped with an ultra-high performance liquid chromatography unit (Thermo Scientific Dionex Ultimate 3000) and a Nanospray Flex Ion-Source (Thermo Scientific). Peptides were loaded on a C18 reversed-phase precolumn (Thermo Scientific) followed by separation on a with 2.4 µm Reprosil C18 resin (Dr. Maisch GmbH) in-house packed picotip emitter tip (diameter 100 µm, 15 cm long from New Objectives) using an gradient from mobile phase A (4 % acetonitrile, 0.1% formic acid) to 40% mobile phase B (80% acetonitrile, 0.1% formic acid) for 60 min followed by a second gradient to 80% B for 30 min with a flow rate 400 nL/min. Run was finished by washout with 99% B for 5 min and reequilibration in 1% B. MS data were recorded by data dependent acquisition Top 10 method selecting the most abundant precursor ions in positive mode for HCD fragmentation. The Full MS scan range was 300 to 2000 m/z with resolution of 70000, and an automatic gain control (AGC) value of 3E6 total ion counts with a maximal ion injection time of 160 ms. Only higher charged ions (2 + ) were selected for MS/MS scans with a resolution of 17500, an isolation window of 2 m/z and an automatic gain control value set to E5 ions with a maximal ion injection time of 150 ms. Selected ions were excluded in a time frame of 20 s following fragmentation event. Fullscan data were acquired in profile and Fragments in centroid mode by Xcalibur software. For data analysis MaxQuant 1.5.3.30 and Perseus 1.5.4.1 were used. The enzyme specificity was set to Trypsin, missed cleavages were limited to 2. Following variable modifications were selected: at N-terminus acetylation (+42.01), oxidation of methionine (+15.99), as fixed modification carbamidomethylation (+57.02) on cysteines. Human reference proteome set from Uniprot (Download 4/2015, 68506 entries) was used to identify peptides and proteins. False discovery rate (FDR) was set to 1 %. Protein group file was uploaded to Perseus and data set was cleaned from reverse identifications and common contaminants. Data were Log2 transformed. Identification were filtered for 4 valid values in at least one group. To enable calculation of ratios between sample and control, missing values were replaced from normal distribution. Positive hits from *p* values (*p* < 0.05) of students *t* test between experimental groups were highlighted. The samples were labeled H1-H5 for HIF1α-AS1 and C1-C5 for the negative control RNA. MaxQuant 1.5.3.30 and Perseus 1.5.4.1 were used to analyze the data.

### RNA immunoprecipitation

1 × 10^7^ HUVECs were used per sample. Nuclei isolation was performed with the truCHIP™ Chromatin Shearing Kit (Covaris, USA) according to the manufacturers protocol without shearing the samples. After pre-clearing with 20 µL DiaMag Protein A and Protein G (Diagenode), 10% of the pre-cleared sample served as input and the lysed nuclei were incubated with the indicated antibody or IgG alone for 12 h at 4 °C. The following antibodies and dilutions were used: Anti-H3-pan (Diagenode, C15200011, 1:200), Anti-dsDNA [35I9 DNA] (Abcam, ab27156, 1:200), Anti-DNA-RNA Hybrid [S9.6] (Kerafast, ENH001, 1:250), Anti-MPP8 (Bethyl, A303-051A-M, 1:250) and Anti-H3K9me3 (Diagenode, SN-146-100, 1:200). The complexes were then incubated with 50 µL DiaMag Protein A and Protein G (Diagenode) beads for 3 h at 4 °C, followed by 4 washing steps in Lysis Buffer from the truCHIP™ Chromatin Shearing Kit (Covaris, USA). In case of RNase treatments, the samples were washed once in TE-buffer and then incubated for 30 min at 37 °C in buffer consisting of 50 mM Tris-HCl pH 7.5-8.0, 150 mM NaCl, 1 mM MgCl_2_ containing 2 µL RNase H per 100 µL buffer. Afterwards the samples were washed in dilution buffer (20 mmol/L Tris/HCl pH 7.4, 100 mmol/L NaCl, 2 mmol/L EDTA, 0.5% Triton X-100, 1 µL Superase In (per 100 µL) and protease inhibitors). Prior to elution, beads were put into a new Eppendorf tube. RNA was extracted with TRIzol (Thermo Fisher) followed by RNA purification with the RNeasy Mini Kit (Qiagen), reverse transcription and qRT-PCR.

For the in vitro RIP assays, the individual RNAs were folded as mentioned above in RNA folding buffer (10 mM Tris pH 7.0, 0.1 M KCl, 10 mM MgCl_2_), and then put on RT for 20 min. The binding reactions with purified MPP8-10xHIS or in vitro translated His-tagged mutants were performed for 2 h at 4 °C in binding buffer (20 mmol/L Tris/HCl pH 8.0, 150 mmol/L KCl, 2 mmol/L EDTA pH 8.0, 5 mmol/L MgCl_2_, 2 µL/mL Superase In and protease inhibitors). After pre-clearing with 20 µL DiaMag Protein A and Protein G (Diagenode), 5% of the pre-cleared sample served as input. The mixture was incubated with Anti-MPP8 (Bethyl, A303-051A-M, 1:250) or Recombinant Anti-6X His tag® antibody [EPR20547] (Abcam, ab213204, ChIP grade, 1:500) for 3 h at 4 °C. The complexes were then incubated with 50 µL DiaMag Protein A and Protein G (Diagenode) beads for 1 h at 4 °C, followed by 4 washing steps (5 min, 4 °C, each) in binding buffer. Elution, RNA extraction and RT-qPCR were performed as mentioned above. RT-qPCR was performed with primers targeting the remaining multiple cloning site (MCS) within the in vitro transcribed sequences before (5ʹ-GTGCTGGATATC TGCAGAATTC-3ʹ) and after (5ʹ-GTGCTGGATATCTGCAGAATTC-3ʹ) the *HIF1α-AS1* sequences.

### Assay for transposase accessibility (ATAC)-sequencing

ATAC-Seq was performed similar to^39^. 100.000 HUVECs were used for ATAC library preparation using Tn5 Transposase from Nextera DNA Sample Preparation Kit (Illumina). Cell pellets were resuspended in 50 µL PBS and mixed with 25 µL TD-Buffer, 2.5 µL Tn5, 0.5 µL 10% NP-40 and 22 µL H_2_O. The mixture was incubated at 37 °C for 30 min followed by 30 min at 50 °C together with 500 mM EDTA pH 8.0 for optimal recovery of digested DNA fragments. 100 µL of 50 mM MgCl_2_ was added for neutralization. The DNA fragments were purified with the MinElute PCR Purification Kit (Qiagen). Amplification of library together with indexing was performed as described elsewhere^72^. Libraries were mixed in equimolar ratios and sequenced on NextSeq500 platform using V2 chemistry and assessed for quality by FastQC. Reaper version 13-100 was employed to trim reads after a quality drop below a mean of Q20 in a window of 5 nt^73^. Only reads above 15 nt were cleared for further analyses. These were mapped versus the hg19 version of the human genome with STAR 2.5.2b using only unique alignments to exclude reads with uncertain arrangement. Reads were further deduplicated using Picard 2.6.0 (Picard: A set of tools (in Java)^74^ for working with next generation sequencing data in the BAM format) to avoid PCR artefacts leading to multiple copies of the same original fragment. The Macs2 peak caller (version 2.1.0)^63^ as employed in punctate mode to accommodate for the range of peak widths typically expected for ATAC-seq. The minimum qvalue was set to −4 and FDR was changed to 0.0001. Peaks overlapping ENCODE blacklisted regions (known misassemblies, satellite repeats) were excluded. Peaks were annotated with the promoter (TSS + /− 5000 nt) of the gene most closely located to the center of the peak based on reference data from GENCODE v19. To compare peaks in different samples, significant peaks were overlapped and unified to represent identical regions. The counts per unified peak per sample were computed with BigWigAverageOverBed (UCSC Genome Browser Utilities, http://hgdownload.cse.ucsc.edu/downloads.html). Raw counts for unified peaks were submitted to DESeq2 (version 1.14.1) for normalization^75^. Spearman correlations were produced to identify the degree of reproducibility between samples using R. To permit a normalized display of samples in IGV, the raw BAM files were normalized for sequencing depth (number of mapped deduplicated reads per sample) and noise level (number of reads inside peaks versus number of reads not inside peaks). Two factors were computed and applied to the original BAM files using bedtools genomecov resulting in normalized BigWig files.

For samples used after siRNA-mediated silencing of MPP8 and *SETDB1* as well as the corresponding LNA GapmeR knockdown of *HIF1α-AS1* or samples from CRISPRa, CRISPRi and LentiCRISPR experiments, the improved OMNI-ATAC protocol^76^ was used and samples were sequenced on a Nextseq2000. The resulting data were trimmed and mapped using Bowtie2^77^. Data were further processed using deepTools^78^. For visualization, the Integrative Genomics Viewer^79^ was used.

### Electrophoretic mobility shift assay (EMSA)

DNA:DNA:RNA triplex samples were analyzed with EMSA using native RNA-PAGE. The samples were prepared with 50% glycerol with 0.3 to 0.5 µM concentration. Native-PAGE gels were prepared using 15% (v/v) polyacrylamide and TA buffer (50 mM Tris/acetate, 50 mM sodium acetate, pH 8.3). Bands were separated at constant power (<1 W) for 5 h and were stained with GelRed® (Biotium, USA) and visualized with the gel documentation imager Gel Doc XR + (Bio-Rad, USA). The following DNA sequences (Dharmacon) were used for triplex target sites: *EPHA2*_3_GA, 5ʹ-AGAGGGTAAGGAGATAGGAGAAACC-3ʹ and *EPHA2*_3_CT, 5ʹ-GGTTTCTCCTATCTCCTTACCCTCT-3ʹ. HIF1α-AS1-TFR2 (TFO2-23) had the following sequence: 5ʹ-GCGGCGGAGGAAAGAGAAAGGAG-3ʹ.

### RNA and DNA Hybridization

By hybridization of the RNA strand to the DNA duplex or DNA hairpin DNA:DNA:RNA triplexes were formed. First the complementary DNA single strands were incubated at 95 °C for 5 min in hybridization buffer (25 mM HEPES, 50 mM NaCl, 10 mM MgCl_2_ (pH 7.4)) and afterwards cooled down to RT. Triplex formation was performed by adding RNA to previously hybridized double stranded DNA for 1 h at 60 °C and then cooled down to RT^13^. For the ^1^H-1D NMR, CD and melting curve experiments, the *HIF1α-AS1*-TFR2 (TFO2-23) sequence 5ʹ-GCGGCGGAGGAAAGAGAAAGGAG-3ʹ (length 23 nt, GC = 50.9%) was used in combination with the DNA sequences listed in Table 2.

**Table 2:** DNA oligos used for ^1^H-1D NMR, CD and melting curve analysis analysis

Name	Sequence (5ʹ−3ʹ)	Size	Genomic location (hg19)
EPHA2 (GA-rich)	GGTTTCTCCTATCTCCTTACCCTCT	25 nt	chr1:16,478,543-16,478,567
EPHA2 (CT-rich)	AGAGGGTAAGGAGATAGGAGAAACC	25 nt	chr1:16,478,543-16,478,567
EPHA2-hairpin	GGTTTCTCCTATCTCCTTACCCTCTTTTTTAGAGGGTAAGGAGATAGGAGAAACC	55 nt	chr1:16,478,543-16,478,567
ADM (CT-rich)	TCTTTCCTCAGCCAC	15 nt	chr11:10,326,521-10,326,535
ADM (GA-rich)	GTGGCTGAGGAAAGA	15 nt	chr11:10,326,521-10,326,535
ADM-hairpin	TCTTTCCTCAGCCACTTTTTGTGGCTGAGGAAAGA	35 nt	chr11:10,326,521-10,326,535

The constructs used were not smaller than the predicted core TFR2:TTS regions.

### CD spectroscopy and melting curve analysis

Circular dichroism spectra were acquired on a Jasco J-810 spectropolarimeter. The measurements were recorded from 210 to 320 nm at 25 °C using 1 cm path length quartz cuvette. CD spectra were recorded on 8 µM samples of each DNA duplex, DNA:RNA heteroduplex and DNA:DNA:RNA-triplex in 25 mM HEPES, 50 mM NaCl, 10 mM MgCl_2_ (pH 7.4). Spectra were acquired with 8 scans and the data was smoothed with Savitzky-Golay filters. Observed ellipticities recorded in millidegree (mdeg) were converted to molar ellipticity [θ] = deg x cm^2^ x dmol^−1^. Melting curves were acquired at constant wavelength using a temperature rate of 1 °C/min in a range from 5 °C to 95 °C. All melting temperature data was converted to normalized ellipticity and evaluated by the following Eq. (1) using SigmaPlot 12.5:

1 $$f=\frac{{{{{{\rm{a}}}}}}}{(1+\exp (-\frac{(x-x0)}{b}))}+\frac{{{{{{\rm{c}}}}}}}{(1+\exp (-\frac{(x-x2)}{d}))}$$

### NMR spectroscopy

All NMR samples were prepared in NMR buffer containing 25 mM HEPES-d18, 50 mM NaCl, 10 mM MgCl_2_ (pH 7.4) with addition of 5 to 10% D_2_O. All samples were internally referenced with 2,2-dimethyl-2-silapentane-5-sulfonate (DSS). The final NMR sample concentrations ranged between 50 µM to 300 µM. NMR spectra were recorded in a temperature range from 278 K to 308 K on Bruker 600, 800, 900 and 950 MHz spectrometers. ^1^H NMR spectra were recorded with jump-return-Echo^80^ and gradient-assisted excitation sculpting^81^ for water suppression. 2D ^1^H,^1^H-NOESY spectra were recorded with jump-return-Echo^80^ water suppression on a Bruker 800 MHz spectrometer at 288 K and mixing times of 150 ms. NMR data were collected, processed and analyzed using TopSpin 3.6.2 (Bruker) and Sparky 3.115^82^.

### Structural modeling

Models of the DNA:DNA:RNA triplex were generated by using the ARIA/CNS software packages^83–85^. To generate and keep the B-form DNA duplex, ample modeling distances and dihedral angle restraints were used. For flexible docking of the RNA on the B-DNA, a starting structure was generated containing a DNA duplex template and an extended RNA molecule. The docking was solely driven by hydrogen-bonds and base-planarity restraints for the triplex. No further restraints were added for the RNA; leaving it fully flexible during the conventional simulated annealing stages with cartesian angle dynamics. In total, 2000 models were generated and the 200 best structures (lowest energy) were used as input for a further refinement in explicit water using the nucleic acid forcefield with OPLS charges and nonbonded parameters^86^. The final ensemble of 20 top-ranked structures was validated and had no violations. Figure production was done by using PyMol 2.5 (Schrödinger, LLC).

### Spheroid outgrowth assay

Spheroid outgrowth assays in HUVEC were performed as described in^87^. Briefly, spheroids were generated by making drops containing 400 HUVECs in a methyl cellulose (20%) (Sigma-Aldrich, M-0512)/culture medium (80%) mixture onto a square petri dish (Greiner Bio-One, 688102). The dish was incubated overnight in upside down direction. Afterwards, spheroids were washed gently with PBS and resuspended in a methyl cellulose (88%)/FCS (12%) mixture and embedded in collagen type I (Corning, 354236) with Medium 199 (Sigma-Aldrich, M0650−100ML). Stimulation of Spheroids was performed with VEGF-A 165 (1 ng/mL) or bFGF (3 ng/mL) for 16 h. Images were generated with an Axiovert135 microscope (Zeiss). Sprout numbers and cumulative sprout lengths were quantified by analysis with the AxioVision software 4.8 (Zeiss).

### Caspase-3/7 activity assay

The Caspase-3/7 activity assays were carried out using 1×10^6^ HUVEC. The assay was performed using SR-FLICA Caspase-3/7 assay Kit (ImmunoChemistry Technologies LLC, 931) following the manufacturer’s instructions. Briefly, cells were washed and a 1:5 dilution of FLICA was added in a dilution of 1:30 to the cell suspension. After an incubation of 1 h, cells were washed three times with buffer provided by the kit, counted and diluted to 3000 cells/µL before measuring emission at 595 nm in a TECAN infinite M200 Pro plate reader using the TECAN i-control 3.7.3.0 software (Männedorf, Switzerland).

### Proximity ligation assay (PLA)

The PLA was performed as described in the manufacturer’s protocol (Duolink II Fluorescence, OLink, Upsalla, Sweden). Briefly, HUVECs were fixed in phosphate buffered formaldehyde solution (4%), permeabilized with Triton X-100 (0.2%), blocked with serum albumin solution (3%) in phosphate-buffered saline, and incubated overnight with Anti-dsDNA [35I9 DNA] (Abcam, ab27156, 1:500), Anti-MPP8 (Bethyl, A303-051A-M, 1:500), Anti-H3K9me3 (Diagenode, SN-146-100, 1:500) or Anti-SETDB1 (Santa Cruz Biotechnology, ESET (G-4): sc-271488, 1:500). Samples were washed and incubated with the respective PLA-probes for 1 h at 37 °C. After washing, samples were ligated for 30 min (37 °C). After an additional washing step, the amplification with polymerase was performed for 100 min (37 °C). The nuclei were stained using DAPI. Images (with Alexa Fluor, 546 nm) were acquired by confocal microscopy (LSM 510, Zeiss) using the ZEN 3.2 software.

### Chromatin Immunoprecipitation

Preparation of HUVEC extracts, crosslinking and isolation of nuclei was performed with the truCHIP™ Chromatin Shearing Kit (Covaris, USA) according to the manufacturers protocol. The procedure was similar to^88^. The lysates were sonified with the Bioruptur Plus (10 cycles, 30 s on, 90 s off, 4 °C; Diagenode, Seraing, Belgium). Cell debris was removed by centrifugation and the lysates were diluted 1:3 in dilution buffer (20 mmol/L Tris/HCl pH 7.4, 100 mmol/L NaCl, 2 mmol/L EDTA, 0.5% Triton X-100 and protease inhibitors). Pre-clearing was done with DiaMag protein A and protein G coated magnetic beads (Diagenode, Seraing, Belgium) for 1 h at 4 °C. As indicated, the samples were incubated over night at 4 °C with the following antibodies and dilutions: Anti-DNA-RNA Hybrid [S9.6] (Kerafast, ENH001, 1:250), Anti-MPP8 (Bethyl, A303-051A-M, 1:250), Anti-SETDB1 (Bethyl, A300-121A, 1:250) and Anti-ZNF638/NP220 (Bethyl, A301-548A-M, 1:250). 5% of the samples served as input. The complexes were collected with 50 µL DiaMag protein A and protein G coated magnetic beads (Diagenode, Seraing, Belgium) for 3 h at 4 °C, washed twice for 5 min with each of the wash buffers 1–3 (Wash Buffer 1: 20 mmol/L Tris/HCl pH 7.4, 150 mmol/L NaCl, 0.1% SDS, 2 mmol/L EDTA, 1% Triton X-100; Wash Buffer 2: 20 mmol/L Tris/HCl pH 7.4, 500 mmol/L NaCl, 2 mmol/L EDTA, 1% Triton X-100; Wash Buffer 3: 10 mmol/L Tris/HCl pH 7.4, 250 mmol/L lithium chloride, 1% Nonidet p-40, 1% sodium deoxycholate, 1 mmol/L EDTA) and finally washed with TE-buffer pH 8.0. In case of RNase treatments, the samples were washed once in TE-buffer and then incubated for 30 min at 37 °C in buffer consisting of 50 mM Tris-HCl pH 7.5-8.0, 150 mM NaCl, 1 mM MgCl_2_ containing 2 µL RNase H or 2 µL RNase A per 100 µL buffer. Elution of the beads was done with elution buffer (0.1 M NaHCO_3_, 1% SDS) containing 1x Proteinase K (Diagenode, Seraing, Belgium) and shaking at 600 rpm for 1 h at 55 °C, 1 h at 62 °C and 10 min at 95 °C. After removal of the beads, the eluate was purified with the QiaQuick PCR purification kit (Qiagen, Hilden, Germany) and subjected to qPCR analysis. As a negative control during qPCR, primer for the promoter of GAPDH were used. The primers are listed in Table 3.

**Table 3:** List of primers for ChIP-qPCR

Name	Forward primer (5ʹ−3ʹ)	Reverse primer (5ʹ−3ʹ)
GAPDH promoter	TGGTGTCAGGTTATGCTGGGCCAG	GTGGGATGGGAGGGTGCTGAACAC
EPHA2 TTS	CAGGTAGCTGCCAATAAGTG	AGGGCTTTACCCTCTGAATC
ADM TTS	CGCGTGGCTGAGGAAAGAAAGG	GCTTTATAAGCGCACGGGTGGG
EPHA2 up (12 kb)	TCAGCTGGGAAGCCACTATG	TTGCTGCTGCTCTGTGAGTC
EPHA2 down (5.7 kb)	CCTCGAATGCATACTCTCAG	CATTCTTGTGCGAGGATGTC
ADM up (2.1 kb)	GGAGGTCAAGGACAGCTAGGC	AGCGAGGTACAGTCGCAGAG
ADM down (3.2 kb)	ACGTGCGGTTTAATAAGTTC	TGGCATCTGCAAACTGTTTC

Number in brackets indicate the approximal distance to the *EPHA2* or *ADM* TTS.

### Triplex domain finder analysis

Triplex formation of *HIF1α-AS1* was predicted using the Triplex Domain Finder 0.13.2 (TDF)^14^ with the human pre-spliced *HIF1α-AS1* sequence (NR_047116.1, gene ID 100750246) to target DNA regions around genes with ATAC-Seq peaks upon *HIF1α-AS1* silencing. For annotation of *HIF1α-AS1* triplex forming regions across DNA triplex target sites, genome version hg19 was used. Boxplots of Fig. 2b show the distribution of triplex prediction from 200 randomizations by shuffling the positions of the same DNA target regions in the genome. Enrichment was given at a p-value <0.05.

### Statistics and reproducibility

Unless otherwise indicated, data are given as means ± standard error of mean (SEM). Calculations were performed with Prism 8.0 or BiAS.10.12. The latter was also used to test for normal distribution and similarity of variance. For multiple group comparisons ANOVA followed by post hoc testing was performed and multiplicity adjusted *p* values were shown, if indicated. Individual statistics of dependent samples were performed by two-tailed Student’s *t* test (paired or unpaired), and if not normally distributed by Mann–Whitney test. *P* values of <0.05 were considered as significant. Unless otherwise indicated, n indicates the number of individual experiments.

### Reporting summary

Further information on research design is available in the Nature Research Reporting Summary linked to this article.

## Supplementary information

Supplementary Information

Description of Additional Supplementary Files

Supplementary Data 1

Supplementary Data 2

Supplementary Data 3

Supplementary Data 4

Supplementary Data 5

Supplementary Data 6

Reporting Summary

## Data Availability

The ATAC-Seq data generated in this study have been deposited in the Sequence Read Archive (SRA) (https://www.ncbi.nlm.nih.gov/sra) under BioProject ID . The CRISPR ATAC-Seq data generated in this study have been deposited in the NCBI’s Gene Expression Omnibus under the GEO Series accession number GSE203252. The mass spectrometry proteomics data about *HIF1α-AS1* interaction partners identified in this study have been deposited to the the ProteomeXchange Consortium via the PRIDE partner repository^89^ with identifier PXD023512. Triplex-Seq data was used from^15^ and is deposited in NCBI GEO under accession number GSE120850. Ensembl hg38 was used for the identification of candidate lncRNAs from the Triplex-Seq data. FANTOM5 ENCODE CAGE expression data was obtained from FANTOM5 website (Gencode v19)^21–23^. ChIP-Seq datasets were taken from ENCODE^90^ and are deposited at NCBI GEO under accession number GSM733673 for HUVEC H3K4me3, for H3K27Ac under accession code GSM733691 and for H3K9Ac under GSM733735. Source data are provided with this paper.

## Source data

Source Data
