# Supplementary material for: HIF1α-AS1 is a DNA:DNA:RNA triplex-forming lncRNA interacting with the HUSH complex
Source: Nat Commun. 2022 Nov 2;13:6563. doi: 10.1038/s41467-022-34252-2 (PMC9630315; doi:10.1038/s41467-022-34252-2)
Supplement: Supplementary file 1 — Supplementary Information [file 41467_2022_34252_MOESM1_ESM.pdf]

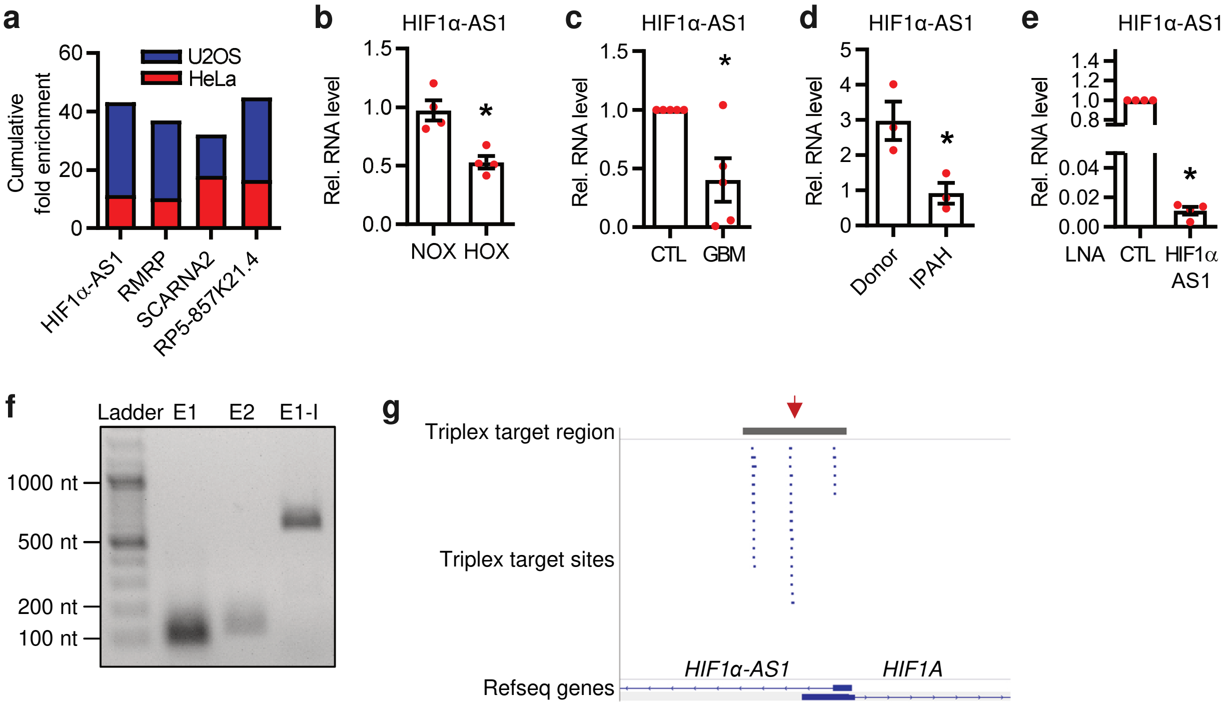

Supplementary Figure 1: a, Cumulative fold enrichment of the four remaining candidates in the U2OS and HeLa S3 Triplex-Seq. **b**, RT-qPCR of *HIF1α-AS1* in paSMCs treated under hypoxic conditions (HOX, 1% O_2) for 24 h. Cells treated under normoxia (NOX) served as basal control. n=4 independent experiments, Unpaired t-test. *(p=0.0048). **c**, RT-qPCR of *HIF1α-AS1* from endothelial cells isolated from glioblastoma (GBM) or adjacent healthy control (CTL) tissue. n=5 biologically independent samples. Paired t-test. *(p=0.0324). **d**, RT-qPCR of *HIF1α-AS1* in paSMCs from control donors (Donor) or patients with idiopathic pulmonary arterial hypertension (IPAH). n=3 biologically independent samples, Unpaired t-test. *(p=0.0299). **e**, RT-qPCR of *HIF1α-AS1* after knockdown with LNA-GapmeRs against *HIF1α-AS1* or an LNA negative control (CTL). n=4 independent experiments, Paired t-test. *(p=<0.0001). **f**, Agarose gel after RT-PCR of Exon1 (E1), Exon2 (E2) or the first 714 nt of the pre-processed *HIF1α-AS1* (E1-I). A representative image of three independent experiments is shown. **g**, Different triplex target sites of *HIF1α-AS1* are shown. Triplex target region is highlighted in grey, triplex target sites are shown in blue. Arrow indicates TTS at the 5'-end. Data are presented as mean values +/- SEM.

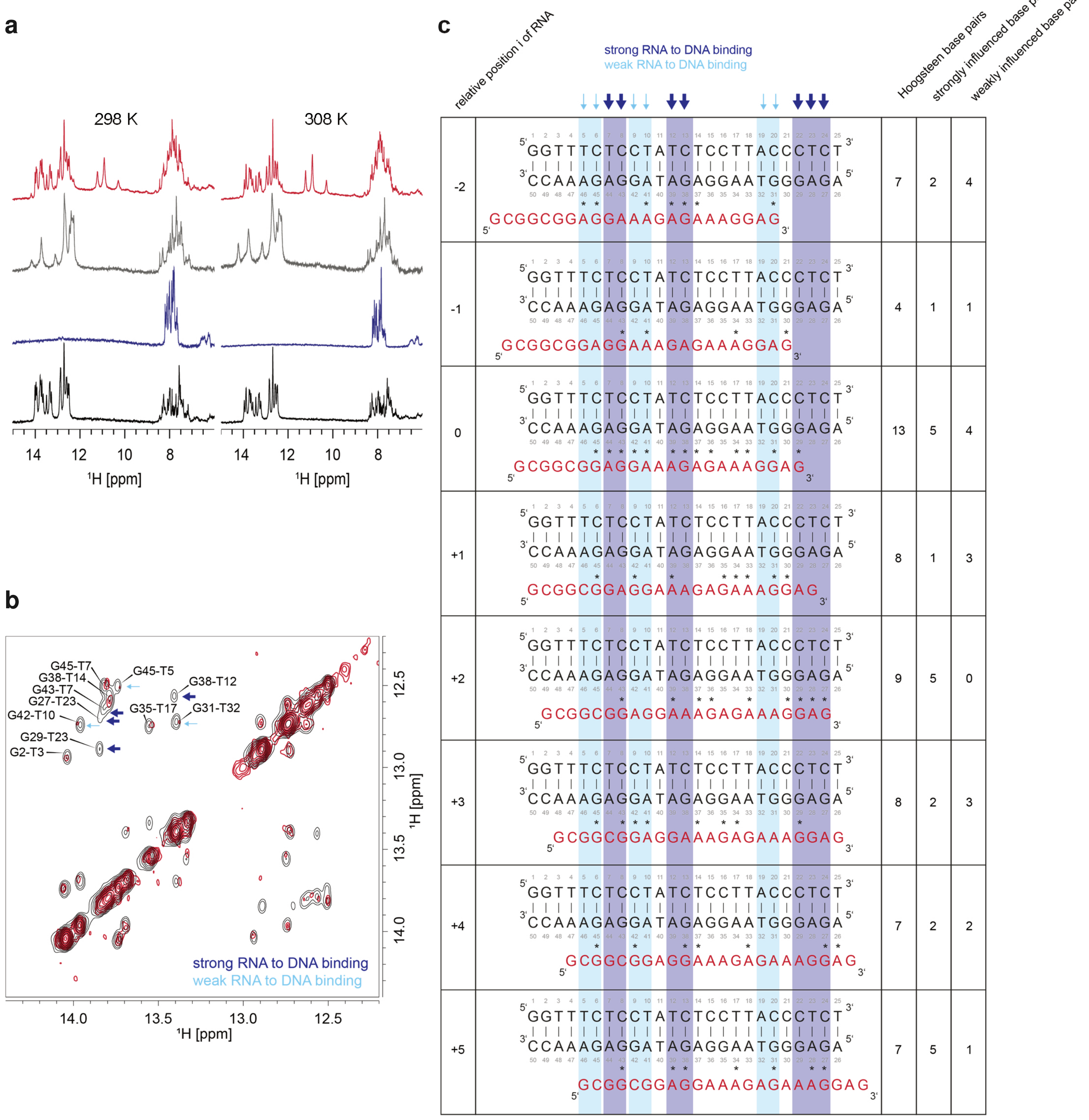

Supplementary Figure 2: NMR analysis of *EPHA2:HIF1α-AS1*_TFR2 triplex and *EPHA2* duplex spectra. **a**, ^{1}H-1D NMR spectra of the *EPHA2* DNA duplex (black), *HIF1α-AS1* TFR2 RNA (blue), heteroduplex (dark grey) and *EPHA2:HIF1α-AS1*-TFR2 triplex (red) at 298 K and 308 K. **b,** Overlay of ^{1}H,^{1}H-NOESY spectrum of *EPHA2* DNA duplex (black) and *EPHA2:HIF1α-AS1*_TFR2 triplex (red) measured at 800 MHz and 288 K in NMR buffer with 5% D_2O. The assigned cross peaks in the NOESY spectra are indicated. Changes in the DNA duplex were quantitatively analyzed. For 7 G- and 6 T-imino protons either a strong (dark blue arrows) or medium (light blue arrows) attenuation of cross peak intensities in the imino-imino region was observed. **c,** Comparison of the predicted Hoogsteen interaction in the triplex with the detected changes in the NOESY spectrum for different positions i of RNA relative to DNA duplex strand.

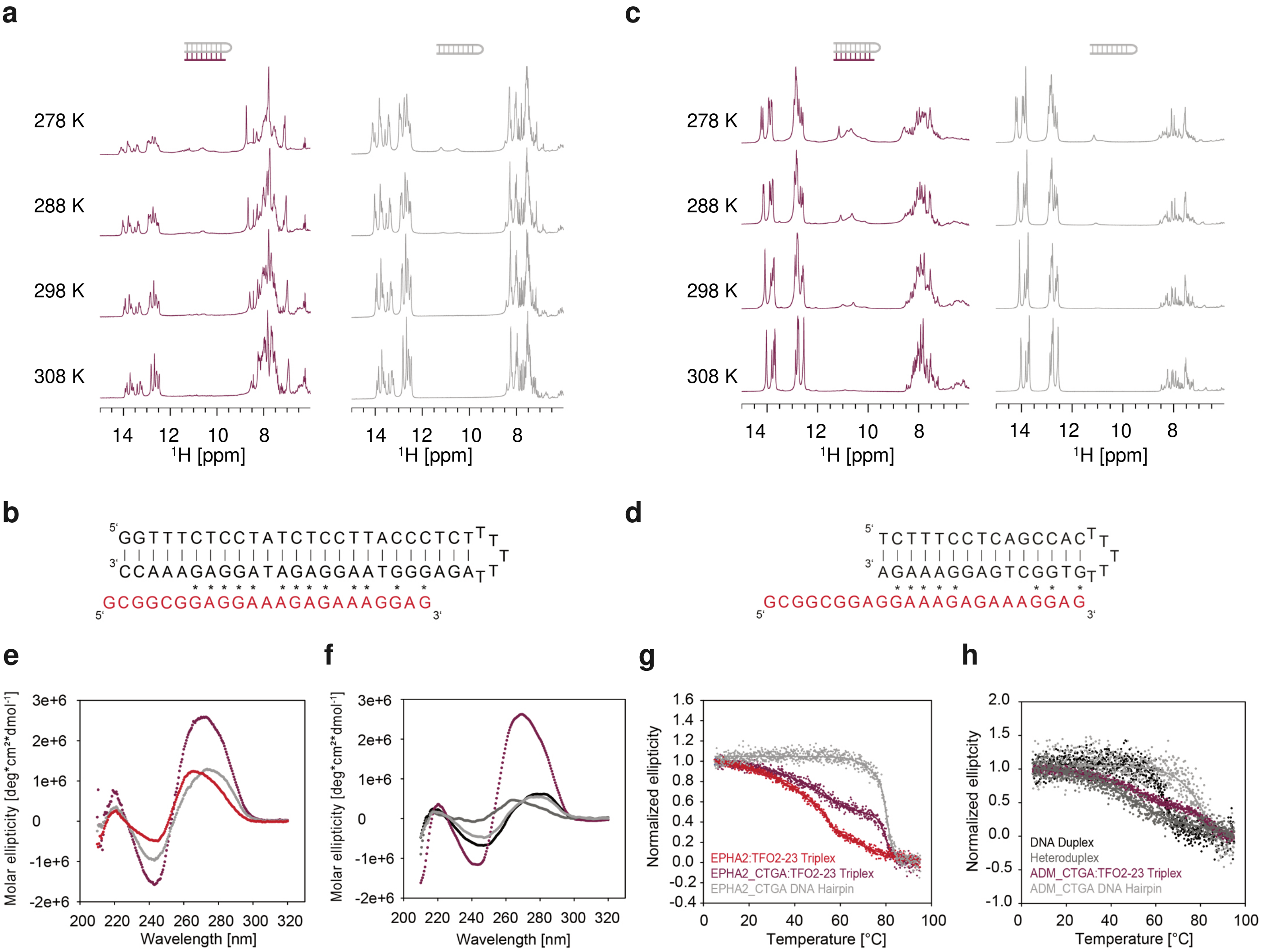

Supplementary Figure 3: *EPHA2* and *ADM* DNA hairpin constructs form *in vitro* DNA:DNA:RNA triplexes with the *HIF1α-AS1* TFR2. a, ^{1}H-1D NMR spectra of the *EPHA2*_CTGA hairpin (grey) and the *EPHA2*_CTGA:*HIF1α-AS1*-TFR2 triplex (dark red) in a temperature range between 278-308 K. **b**, Sequence of *EPHA2*_CTGA hairpin DNA (black) and *HIF1α-AS1*-TFR2 RNA (red). Watson-Crick base pairing is indicated with | and the Hoogsteen base pairing is indicated with *. **c**, ^{1}H-1D NMR spectra of the *ADM*_CTGA hairpin (grey) and the *ADM*_CTGA:*HIF1α-AS1*-TFR2 triplex (dark red) in a temperature range between 278-308 K. **d**, Sequence of *ADM*_CTGA hairpin DNA (black) and *HIF1α-AS1*-TFR2 RNA (red). Watson-Crick base pairing is indicated with | and the Hoogsteen base pairing is indicated with *. **e**, Circular dichroism spectra of the *EPHA2:HIF1α-AS1*-TFR2 (TFO2-23) triplex (red), the *EPHA2*_CTGA hairpin alone (light grey) and the *EPHA2*_CTGA:*HIF1α-AS1*-TFR2 (TFO2-23) triplex (dark red) measured at 298 K. **f**, Circular dichroism spectra of the *ADM* duplex (black), the heteroduplex (dark grey), the *ADM*_CTGA hairpin alone (light grey) and the *ADM*_CTGA:*HIF1α-AS1*-TFR2 (TFO2-23) triplex (dark red) measured at 298 K. **g**, Thermal melting of the *EPHA2:HIF1α-AS1*-TFR2 (TFO2-23) triplex (red), the *EPHA2*_CTGA hairpin (light grey) and *EPHA2*_CTGA:*HIF1α-AS1*-TFR2 (TFO2-23) (dark red). **h**, Thermal melting of the *ADM* duplex (black), the heteroduplex (dark grey), the *ADM*_CTGA hairpin (light grey) and *ADM*_CTGA:*HIF1α-AS1*-TFR2 (TFO2-23) (dark red).

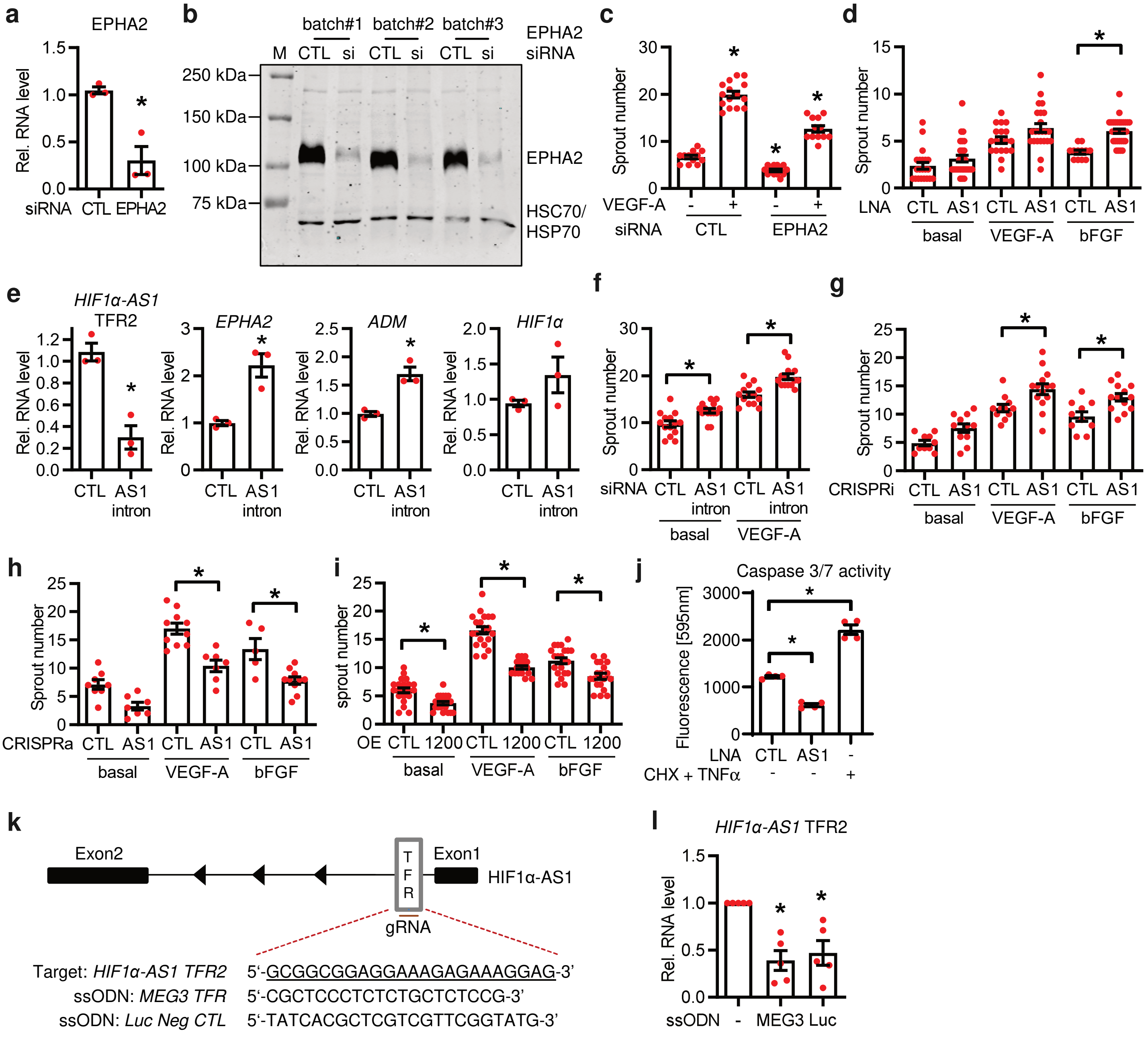

Supplementary Figure 4: a, RT-qPCR after siRNA-mediated knockdown of *EPHA2*. Expression levels of *EPHA2* are shown. Scrambled siRNA (CTL) served as negative control. n=3 independent experiments, Unpaired t-test. *(p=0.0083). **b**, Western blot with (si) or without (CTL) siRNA-mediated knockdown of *EPHA2* in three different batches of HUVEC. EPHA2 and HSC70/HSP70 antibodies were used. M, marker **c**, Quantification of the sprout numbers from the spheroid assay seen in Fig. 4d. One-Way ANOVA with Bonferroni test. CTL-VEGF-A, n=12; CTL+VEGF-A, n=15; EPHA2-VEGF-A, n=13; EPHA2+VEGF-A, n=12. *CTL-/+VEGF-A(p=<0.0001), *EPHA2-/+VEGF-A(p=<0.0001), *CTL/EPHA2+VEGF-A(p=<0.0001), *CTL/EPHA2-VEGF-A(p=0.0054). **d**, Quantification of the sprout numbers from the spheroid assay seen in Fig. 4f. One-Way ANOVA with Bonferroni test. CTL-basal(n=21), AS1-basal(n=26), CTL+VEGF-A(n=19), AS1+VEGF-A(n=23), CTL+bFGF(n=12), AS1+bFGF(n=32). *(p=0.0021). **e**, RT-qPCR after siRNA-mediated knockdown targeting the *HIF1α-AS1* intron. Expression levels of *HIF1α-AS1* TFR2, *EPHA2*, *ADM* and *HIF1α* are shown. Scrambled siRNA (CTL) served as negative control. n=3 independent experiments, Unpaired t-test. *TFR2(p=0.0043), *EPHA2(p=0.0082), *ADM(p=0.0053). **f-i**, Quantification of the sprout numbers from the spheroid outgrowth assays with siRNA targeting the *HIF1α-AS1* intron (f, n=14), with CRISPRi (g, CTL-basal(n=10), AS1-basal(n=11), CTL+VEGF-A(n=11), AS1+VEGF-A(n=14), CTL+bFGF(n=10), AS1+bFGF(n=13)), with CRISPRa (h, CTL-basal(n=8), AS1-basal(n=7), CTL+VEGF-A(n=10), AS1+VEGF-A(n=7), CTL+bFGF(n=5), AS1+bFGF(n=10)) or after overexpression (i, CTL-basal(n=21), 1200-basal(n=22), CTL+VEGF-A(n=20), 1200+VEGF-A(n=18), CTL+bFGF(n=21), 1200+bFGF(n=19)) of the first 1200 nt of the *HIF1α-AS1* gene (included TFR2, named as 1200). One-Way ANOVA with Bonferroni test. f: *basal(p=0.0094), *VEGF-A(p=0.0002); g: *VEGF-A(p=0.0324), *bFGF(p=0.0407); h: *VEGF-A(p=0.0002), *bFGF(p=0.0071); i: *basal(p=0.0095), *VEGF-A(p=<0.0001), *bFGF(p=0.0011). **j**, Caspase-3/7 activity assay with or without LNA-GapmeR-mediated knockdown of *HIF1α-AS1* in HUVEC. Cycloheximide (CHX, 50 µg/mL) and TNF-α (20 ng/mL) stimulation for 16 h served as positive control. n=4 independent experiments, One-Way ANOVA with Bonferroni post-hoc test. *CTL/AS1(p=0.0026), *CTL/CHX+TNF-α(p=0.003). **k**, Scheme of the CRISPR ArciTect approach. TFR2 of *HIF1α-AS1* (underlined) was targeted with Cas9/gRNA and replaced with ssODNs including *MEG3* TFR or a DNA fragment of luciferase negative control. **l**, Relative RNA level of *HIF1α-AS1* TFR2 after a ssODN-mediated replacement of the TFR2 within *HIF1α-AS1* with the TFR of MEG3 or a DNA fragment of a luciferase negative control (Luc). -, no ssODN used. n=5 independent experiments, Paired t-test. *MEG3(p=0.0006), *Luc(p=0.0016). Data are presented as mean values +/- SEM.

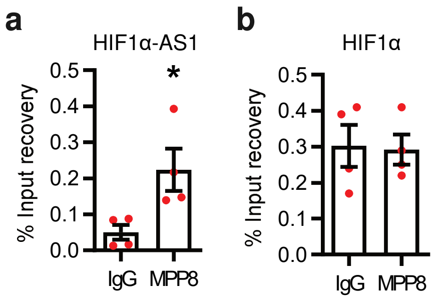

c

#	Protein region	RNA region	Interaction Propensity	Discriminative Power	Normalized Score
1	51-102	26-77	18.73	50	5.41
2	51-102	27-78	17.80	47	5.17
4	726-777	26-77	15.90	42	4.67
3	51-102	51-102	15.91	42	4.67
5	726-777	27-78	14.92	40	4.41
6	51-102	52-103	14.56	40	4.32
7	360-411	26-77	14.53	40	4.31
8	735-786	26-77	13.60	37	4.06
9	360-411	27-78	13.31	37	3.99
10	735-786	27-78	13.01	37	3.91

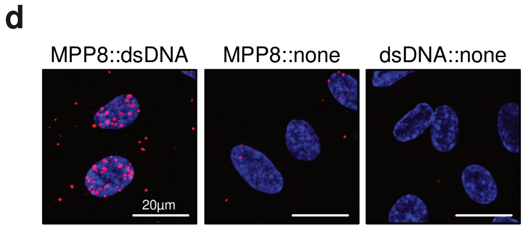

Supplementary Figure 5: a&b, RIP with MPP8 antibodies and qPCR for *HIF1α-AS1* (a) or HIF1α (b). IgG served as negative control. n=4 independent experiments, Mann-Whitney t-test. *(p=0.0286). **c**, Binding propensity of MPP8 and *HIF1α-AS1* calculated with *cat*RAPID. **d**, Proximity ligation assay of HUVECs with antibodies against MPP8 and dsDNA. The individual antibody alone served as negative control. Red dots indicate polymerase amplified interaction signals. Scale bar indicates 20 µm. Images were representative of three independent experiments. Data are presented as mean values +/- SEM.

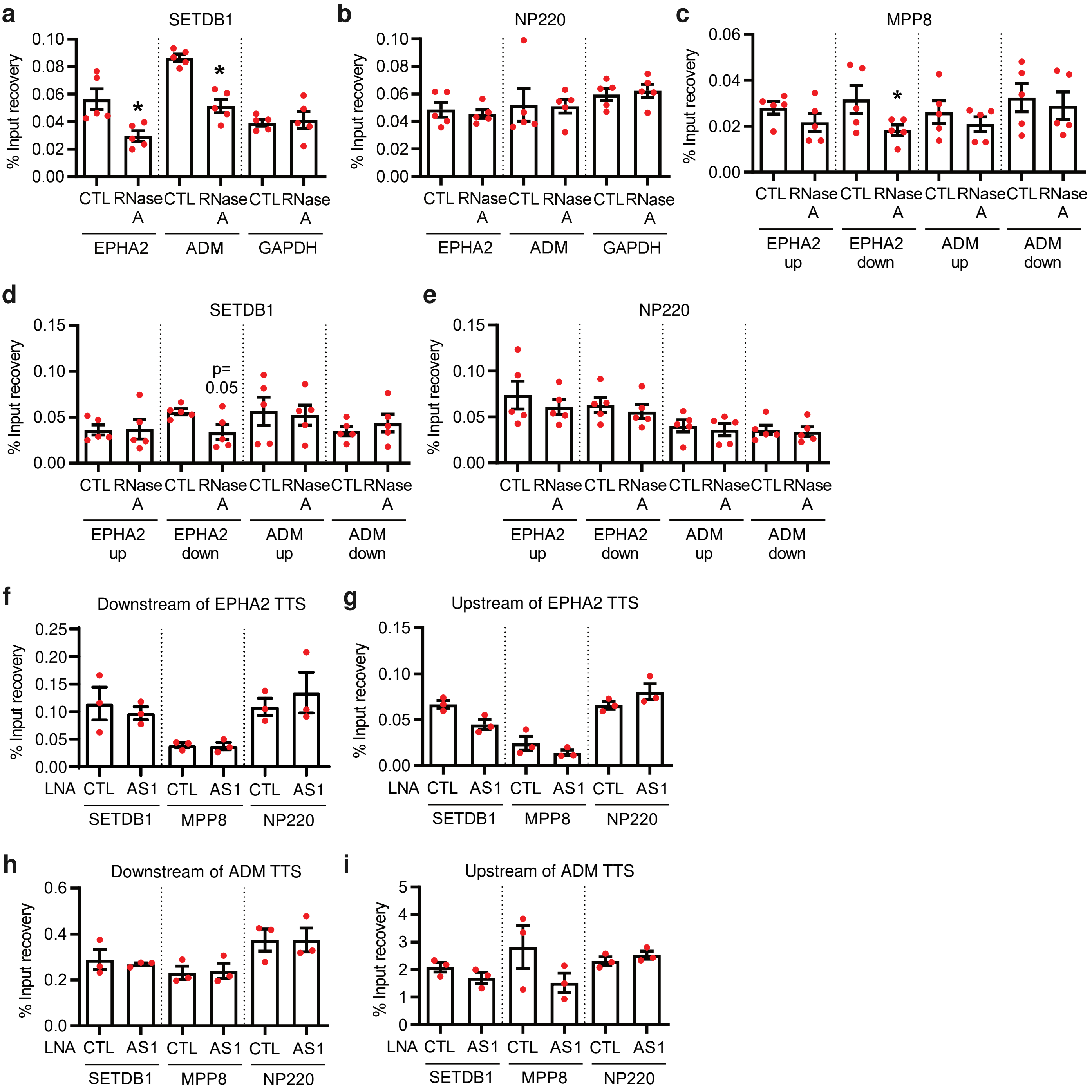

Supplementary Figure 6: a&b, ChIP with SETDB1 (a) or NP220 (b) antibodies with or without RNase A treatment and qPCR for the triplex target sites (TTS) of *EPHA2* and *ADM*. Primers against a promoter sequence of GAPDH served as negative control. n=5 independent experiments, paired t-test. a: *EPHA2(p=0.0263), *ADM(p=0.0102). **c-e**, ChIP with MPP8 (c), SETDB1 (d) or NP220 (e) antibodies with or without RNase A treatment. QPCR was performed for a region downstream (down) or upstream (up) of *EPHA2* or of *ADM* TTS. n=5 independent experiments, paired t-test. c: *(p=0.0279). **f-i**, ChIP with antibodies against SETDB1, MPP8 or NP220 in HUVECs treated with (AS1) or without (CTL) LNA GapmeRs against *HIF1α-AS1*. QPCR was performed for a region downstream or upstream of *EPHA2* TTS (f, g) or of *ADM* TTS (h, i). n=3 independent experiments, paired t-test. Data are presented as mean values +/- SEM.

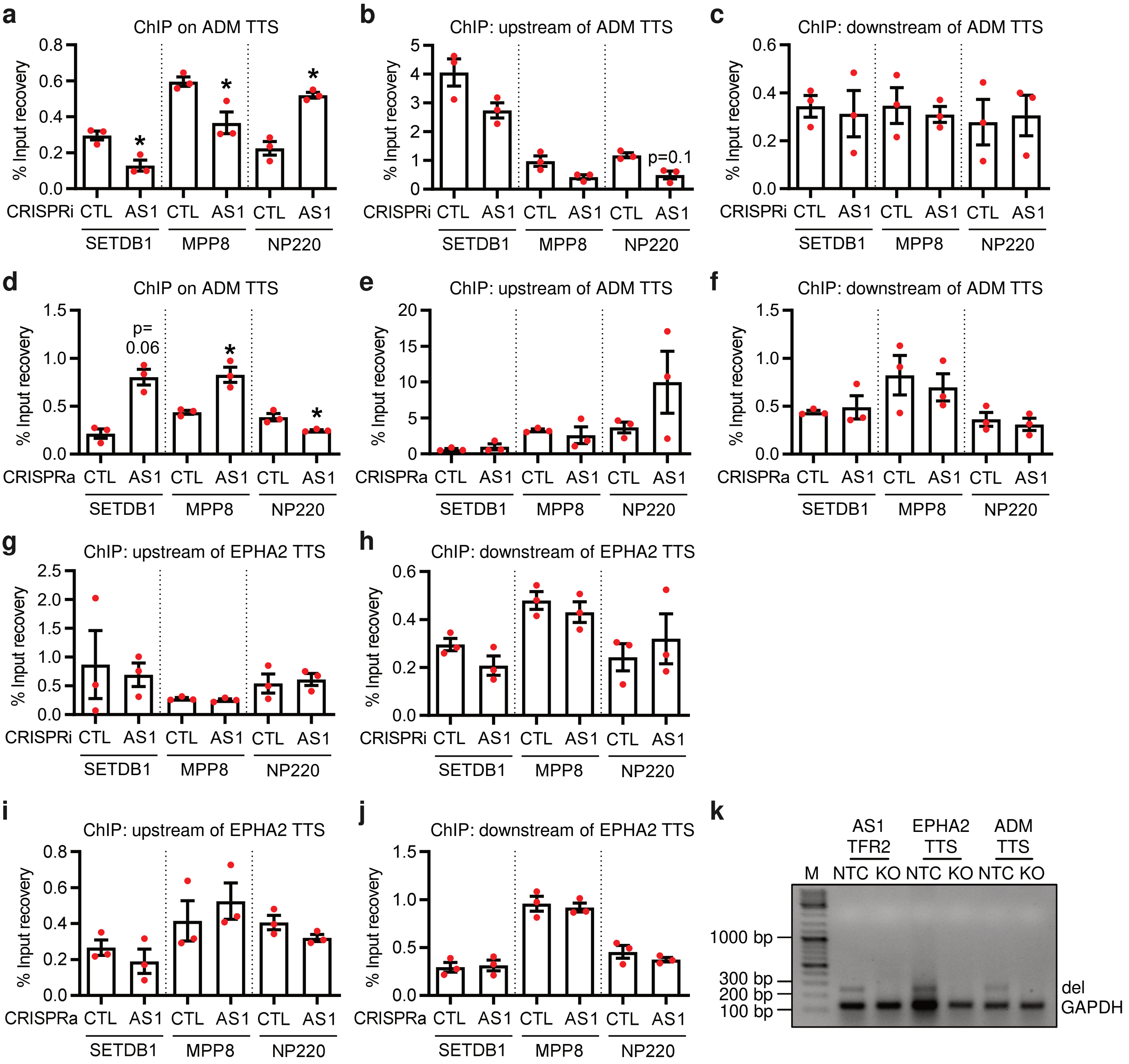

Supplementary Figure 7: a-c, ChIP with SETDB1, MPP8 or NP220 antibodies after CRISPRi for *HIF1α-AS1* and qPCR for *ADM* TTS (a) or a region upstream (b) or downstream (c) of *ADM* TTS. n=3 independent experiments, paired t-test. a: *SETDB1(p=0.0406), *MPP8(p=0.0479), *NP220(p=0.0367). **d-f**, ChIP with SETDB1, MPP8 or NP220 antibodies after CRISPRa for *HIF1α-AS1* and qPCR for *ADM* TTS (d) or a region upstream (e) or downstream (f) of *ADM* TTS. n=3 independent experiments, paired t-test. d: *MPP8(p=0.0319), *NP220(p=0.0239). **g-h**, ChIP with SETDB1, MPP8 or NP220 antibodies after CRISPRi for *HIF1α-AS1* and qPCR for a region upstream (g) or downstream (h) of *EPHA2* TTS. n=3 independent experiments, paired t-test. **i-j**, ChIP with SETDB1, MPP8 or NP220 antibodies after CRISPRa for *HIF1α-AS1* and qPCR for a region upstream (i) or downstream (j) of *EPHA2* TTS. n=3 independent experiments, paired t-test. **k**, PCR of genomic DNA after lentiviral CRISPR/Cas9-mediated deletion (KO) of HIF1α-AS1 TFR2, EPHA2 TTS and ADM TTS in HUVEC. A non-targeting control gRNA (NTC) served as negative control. Primers targeting the deleted region (del) were used together with primers for GAPDH (loading control). A representative image of three replicates is shown. Data are presented as mean values +/- SEM.

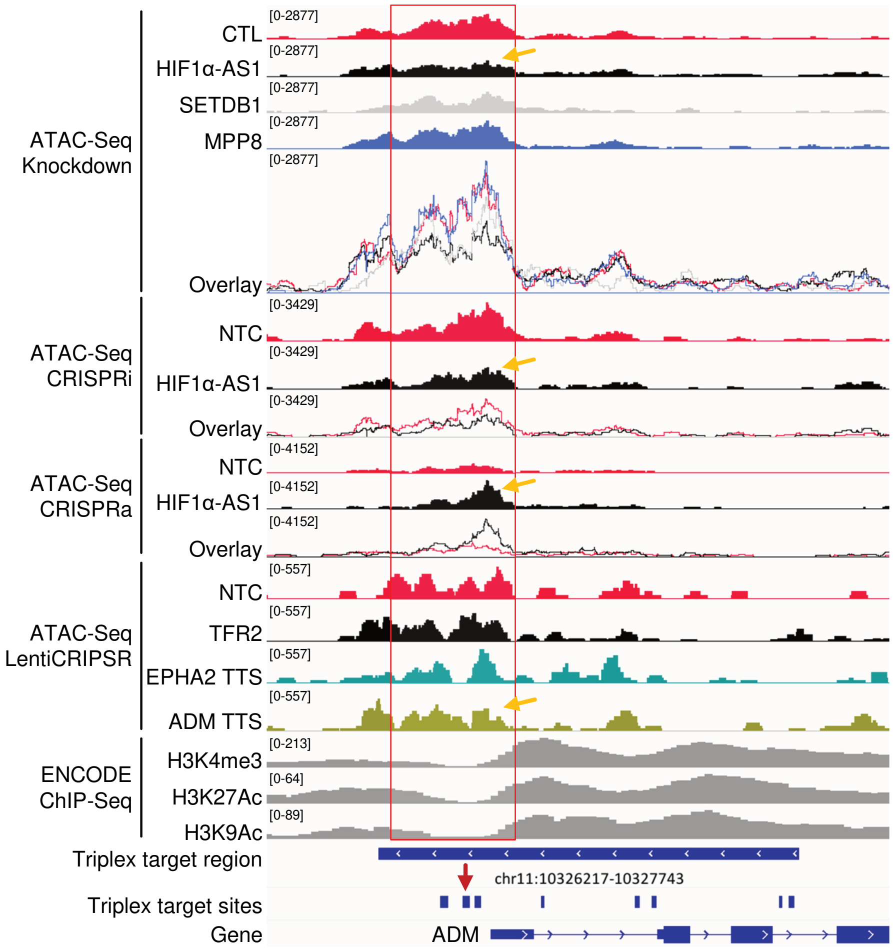

Supplementary Figure 8: Genome tracks for ADM of ATAC-Seq in HUVECs separately and as an overlay after knockdown of HIF1α-AS1 (black), SETDB1 (grey), MPP8 (blue) or the negative control (red), after CRISPRi and CRISPRa of HIF1α-AS1 or after LentiCRISPR-mediated deletions of HIF1α-AS1 TFR2, EPHA2 TTS or ADM TTS. ChIP-Seq data (H3K4me3, H3K27Ac, H3K9Ac) in HUVECs was derived from ENCODE. Numbers in square brackets indicate data range values. Red arrow indicates the TTS analyzed in this study, orange arrows indicate strong changes. The red box indicates the relevant location. NTC, non-targeting control.

Source Data file containing uncropped blots and gels from Supplementary Figures

Supplementary Figure 1f

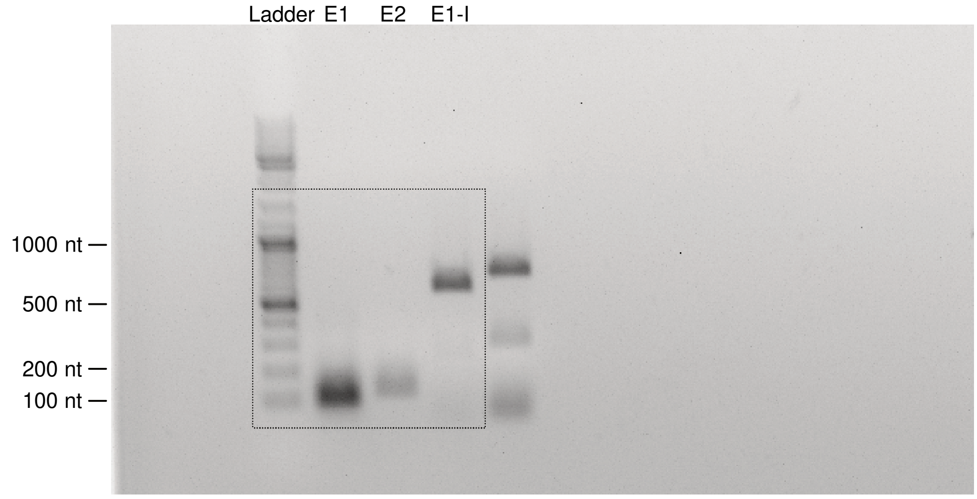

dashed line: figure shown in manuscript

Supplementary Figure 4b

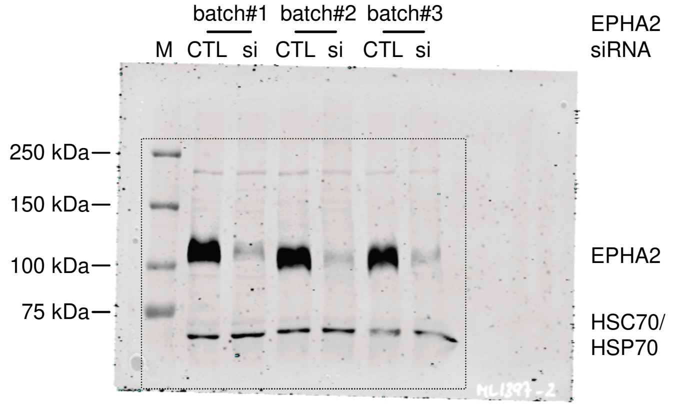

dashed line: figure shown in manuscript

Supplementary Figure 7k

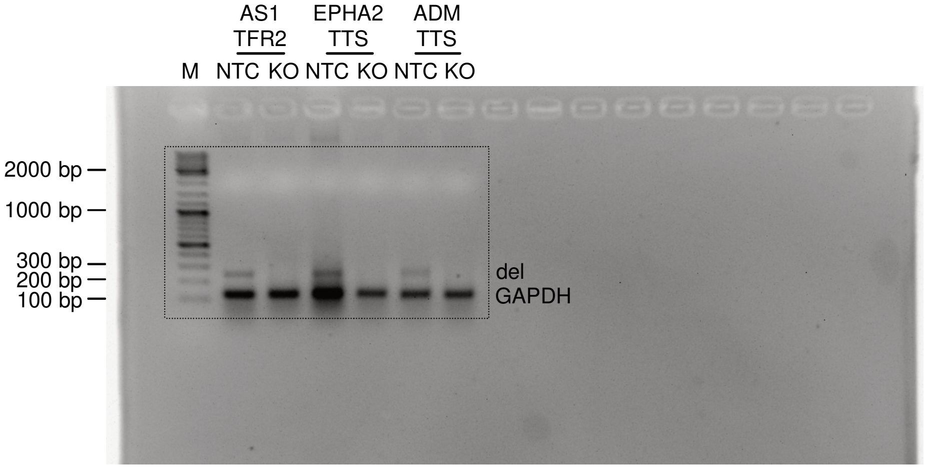

dashed line: figure shown in manuscript
