# Supplementary material for: HIF1α-AS1 is a DNA:DNA:RNA triplex-forming lncRNA interacting with the HUSH complex
Source: Nat Commun. 2022 Nov 2;13:6563. doi: 10.1038/s41467-022-34252-2 (PMC9630315; doi:10.1038/s41467-022-34252-2)
Supplement: Supplementary file 2 — Description of Additional Supplementary Files [file 41467_2022_34252_MOESM2_ESM.pdf]

Description of Additional Supplementary Files

File Name: Supplementary Data 1
Description: Triplex-Seq HeLa S3 lncRNA regions

File Name: Supplementary Data 2
Description: Triplex-Seq U2OS lncRNA regions

File Name: Supplementary Data 3
Description: List of TTS for TFR1

File Name: Supplementary Data 4
Description: List of TTS for TFR2

File Name: Supplementary Data 5
Description: List of TTS for TFR3

File Name: Supplementary Data 6
Description: Interaction partners of HIF1α-AS1, n=5. Abbreviations: H, HIF1α-AS1; C, Control RNA.
