# Supplementary material for: HIF1α-AS1 is a DNA:DNA:RNA triplex-forming lncRNA interacting with the HUSH complex
Source: Nat Commun. 2022 Nov 2;13:6563. doi: 10.1038/s41467-022-34252-2 (PMC9630315; doi:10.1038/s41467-022-34252-2)
Supplement: Supplementary file 9 — Reporting Summary [file 41467_2022_34252_MOESM9_ESM.pdf]

nature portfolio

Corresponding author(s): Ralf P. Brandes, Harald Schwalbe

Last updated by author(s): Oct 9, 2022

Reporting Summary

Nature Portfolio wishes to improve the reproducibility of the work that we publish. This form provides structure for consistency and transparency in reporting. For further information on Nature Portfolio policies, see our Editorial Policies and the Editorial Policy Checklist.

Statistics

For all statistical analyses, confirm that the following items are present in the figure legend, table legend, main text, or Methods section.

n/a	Confirmed	
☐	☒	The exact sample size (*n*) for each experimental group/condition, given as a discrete number and unit of measurement
☐	☒	A statement on whether measurements were taken from distinct samples or whether the same sample was measured repeatedly
☐	☒	The statistical test(s) used AND whether they are one- or two-sided<br>*Only common tests should be described solely by name; describe more complex techniques in the Methods section.*
☐	☒	A description of all covariates tested
☐	☒	A description of any assumptions or corrections, such as tests of normality and adjustment for multiple comparisons
☐	☒	A full description of the statistical parameters including central tendency (e.g. means) or other basic estimates (e.g. regression coefficient) AND variation (e.g. standard deviation) or associated estimates of uncertainty (e.g. confidence intervals)
☐	☒	For null hypothesis testing, the test statistic (e.g. F, t, r) with confidence intervals, effect sizes, degrees of freedom and P value noted<br>*Give P values as exact values whenever suitable.*
☒	☐	For Bayesian analysis, information on the choice of priors and Markov chain Monte Carlo settings
☒	☐	For hierarchical and complex designs, identification of the appropriate level for tests and full reporting of outcomes
☒	☐	Estimates of effect sizes (e.g. Cohen's *d*, Pearson's *r*), indicating how they were calculated

Our web collection on statistics for biologists contains articles on many of the points above.

Software and code

Policy information about availability of computer code

Data collection	Triplex domain finder software 0.13.2 (Kuo et al., 2019, NAR, doi: 10.1093/nar/gkz037);<br>Testing of noncoding probability with online tools CPAT 3.0.0 (Wang et al., 2013, NAR, doi: 10.1093/nar/gkt006) and CPC2 (Kang et al., 2017, NAR, doi: 10.1093/nar/gkx428).<br>CRISPOR algorithm 5.01 was used to design gRNAs for LentiCRISPRv2 (Haeussler et al., 2016, Genome Biology, doi: 10.1186/s13059-016-1012-2).
Data analysis	Agilent Aria 1.7, Image Studio 5.2, Reaper Version 13-100, STAR 2.5.2b, Picard 2.6.0, Macs2 peak caller (version 2.1.0), Bowtie2, BigWigAverageOverBed (UCSC Genome Browser Utilities, [http://hgdownload.cse.ucsc.edu/downloads.html], DESeq2 (version 1.14.1), MaxQuant 1.5.3.30, Perseus 1.5.4.1, TECAN i-control 3.7.3.0, GraphPad Prism 8, Axiovision 4.8, ZEN 3.2, SigmaPlot 12.5, TopSpin 3.6.2, PyMol 2.5

For manuscripts utilizing custom algorithms or software that are central to the research but not yet described in published literature, software must be made available to editors and reviewers. We strongly encourage code deposition in a community repository (e.g. GitHub). See the Nature Portfolio guidelines for submitting code & software for further information.

Data

Policy information about availability of data

All manuscripts must include a data availability statement. This statement should provide the following information, where applicable:

- Accession codes, unique identifiers, or web links for publicly available datasets
- A description of any restrictions on data availability
- For clinical datasets or third party data, please ensure that the statement adheres to our policy

Data availability
The ATAC-Seq data generated in this study have been deposited in the NCBI SRA and Bioproject database under accession code PRJNA765209 [https://www.ncbi.nlm.nih.gov/bioproject/PRJNA765209/].
The CRISPR ATAC-Seq data generated in this study have been deposited in the NCBI GEO database under accession code GSE203252 [https://www.ncbi.nlm.nih.gov/geo/query/acc.cgi?acc=GSE203252].
The data about HIF1α-AS1 interaction partners identified with mass spectrometry generated in this study have been deposited in PRIDE ProteomeXchange (title: Identification of interacting proteins of lncRNA HIF1α-AS1) under accession code PXD023512 [https://www.ebi.ac.uk/pride/archive/projects/PXD023512].

Publicly available datasets used
Triplex-Seq data was used from Sentürk et al. (doi: 10.1093/nar/gky1305) and deposited in NCBI GEO (GSE120850) [https://www.ncbi.nlm.nih.gov/geo/query/acc.cgi?acc=GSE120850]. Ensembl hg38 [https://www.ensembl.org/Human/Search/Results?q=;site=ensembl;facet_species=Human] was used for the identification of candidate lncRNAs from the Triplex-Seq data. FANTOM5 ENCODE CAGE expression data was obtained from FANTOM5 website [https://fantom.gsc.riken.jp/5/] (Gencode v19) (doi: 10.1038/nature13182; doi: 10.1186/s13059-014-0560-6; doi: 10.1038/sdata.2017.112). ChIP-Seq datasets for HUVEC H3K4me3 (GSM733673) [https://www.ncbi.nlm.nih.gov/geo/query/acc.cgi?acc=GSM733673], H3K27Ac (GSM733691) [https://www.ncbi.nlm.nih.gov/geo/query/acc.cgi?acc=GSM733691] and H3K9Ac (GSM733735) [https://www.ncbi.nlm.nih.gov/geo/query/acc.cgi?acc=GSM733735] were taken from ENCODE (doi: 10.1038/nature11247) and are deposited at NCBI GEO.

Triplex domain finder analysis
Triplex formation of HIF1α-AS1 was predicted using the Triplex Domain Finder 0.13.2 (TDF) (doi: 10.1093/nar/gkz037) with the human pre-spliced HIF1α-AS1 sequence (NR_047116.1, gene ID 100750246) to target DNA regions around genes with ATAC-Seq peaks upon HIF1α-AS1 silencing. For annotation of HIF1α-AS1 triplex forming regions across DNA triplex target sites, genome version hg19 was used. Randomization was performed for 200 times. Enrichment was given at a p-value <0.05.

Human research participants

Policy information about studies involving human research participants and Sex and Gender in Research.

Reporting on sex and gender	Sex and gender were not considered and evaluated in this study.
Population characteristics	Population characteristics of the human research participants were not evaluated. Instead, material was obtained from a small number of patients for RT-qPCR analysis with the indicated medical record regardless of age, gender and ethnicity.
Recruitment	This study did not involve active recruitment of participants.
Ethics oversight	Pulmonary hypertension:<br>The study protocol for tissue donation from human idiopathic pulmonary hypertension patients was approved by the ethics committee (Ethik Kommission am Fachbereich Humanmedizin der Justus Liebig Universität Giessen) of the University Hospital Giessen (Giessen, Germany) in accordance with national law and with Good Clinical Practice/International Conference on Harmonisation guidelines. Written informed consent was obtained from each individual patient or the patient's next of kin (AZ 31/93, 10/06, 58/15) (Savai et al., Nature Medicine, 2014, doi: 10.1038/nm.3695).<br><br>Glioblastoma (GBM):<br>Studies for human glioblastoma were covered by an ethics statement according to the guidelines of the University of Frankfurt, whose approval number for autopsy material is GS-249/11 and for resection material GS-04/09 as published already (Leisegang et al., 2017, Circulation, doi: 10.1161/CIRCULATIONAHA.116.026991).

Note that full information on the approval of the study protocol must also be provided in the manuscript.

Field-specific reporting

Please select the one below that is the best fit for your research. If you are not sure, read the appropriate sections before making your selection.

☒ Life sciences ☐ Behavioural & social sciences ☐ Ecological, evolutionary & environmental sciences

For a reference copy of the document with all sections, see nature.com/documents/nr-reporting-summary-flat.pdf

Life sciences study design

All studies must disclose on these points even when the disclosure is negative.

Sample size	Sample sizes were chosen based on previous experiences and studies with similar types of experiments (PMID: 31222221; PMID: 28351900; PMID: 32341350).
Data exclusions	No data was excluded.
Replication	All results were reproduced in at least three independent replicates including all successful attempts; sequencing data experiments were performed as indicated.
Randomization	Samples were collected to study groups by treatment conditions. Randomization was not applied.
Blinding	Blinding was not relevant due to objective readouts (ATAC-Seq, ChIP-qPCR, RT-qPCR). Investigators were not blinded.

Reporting for specific materials, systems and methods

We require information from authors about some types of materials, experimental systems and methods used in many studies. Here, indicate whether each material, system or method listed is relevant to your study. If you are not sure if a list item applies to your research, read the appropriate section before selecting a response.

Materials & experimental systems

n/a	Involved in the study	
☐	☒	Antibodies
☐	☒	Eukaryotic cell lines
☒	☐	Palaeontology and archaeology
☒	☐	Animals and other organisms
☒	☐	Clinical data
☒	☐	Dual use research of concern

Methods

n/a	Involved in the study	
☒	☐	ChIP-seq
☒	☐	Flow cytometry
☒	☐	MRI-based neuroimaging

Antibodies

Antibodies used

Anti-H3-pan (Diagenode, C15200011)
Anti-dsDNA [35I9 DNA] (Abcam, ab27156)
Anti-DNA-RNA Hybrid [S9.6] (Kerafast, ENH001)
Anti-EPHA2 (Bethyl, A302-025-M)
Anti-GAPDH (Sigma, G8795)]
Anti-HSC70/HSP70 (Enzo Life Sciences, ADI-SPA-820)
Anti-NONO (Bethyl, A300-587A)
Anti-MPP8 (Bethyl, A303-051A-M)
Recombinant Anti-6X His tag® antibody [EPR20547] (Abcam, ab213204, ChIP grade)
Anti-H3K9me3 (Diagenode, SN-146-100)
Anti-SETDB1 (Bethyl, A300-121A)
Anti-SETDB1 (Santa Cruz Biotechnology, ESET (G-4): sc-271488)
Anti-ZNF638/NP220 (Bethyl, A301-548A-M)
Dilutions used for the individual experiments can be found in the methods section.

Validation

All antibodies were purchased from commercial vendors, who provide validation information on their website.
Anti-H3-pan (Diagenode, C15200011): Validation data for ChIP, WB and IF existing on the website [https://www.diagenode.com/en/p/h3pan-monoclonal-antibody-classic-50-mg-100-ml]
Anti-dsDNA [35I9 DNA] (Abcam, ab27156): Validation data on the website [https://www.abcam.com/goat-mouse-igg-hl-hrp-ab205719.html] and used in publications ([https://www.nature.com/articles/s41467-022-31801-7], [https://doi.org/10.1093/nar/gky1305])
Anti-DNA-RNA Hybrid [S9.6] (Kerafast, ENH001): Validation data on the website [https://www.kerafast.com/productgroup/432/anti-dna-rna-hybrid-s96-antibody]
Anti-EPHA2 (Bethyl, A302-025-M): Validation data on the website [https://www.thermofisher.com/antibody/product/EphA2-Antibody-Polyclonal/A302-025A]
Anti-GAPDH (Sigma, G8795): Validation data on the website [https://www.sigmaaldrich.com/DE/en/product/sigma/g8795]
Anti-HSC70/HSP70 (Enzo Life Sciences, ADI-SPA-820): Validation data on the website [https://www.enzolifesciences.com/ADI-SPA-820/hsc70-hsp70-monoclonal-antibody-n27f3-4/]
Anti-NONO (Bethyl, A300-587A): Validation data on the website [https://www.thermofisher.com/antibody/product/NONO-Antibody-Polyclonal/A300-587A]
Anti-MPP8 (Bethyl, A303-051A-M): [https://www.thermofisher.com/antibody/product/MPP8-Antibody-Polyclonal/A303-051A]
Recombinant Anti-6X His tag® antibody [EPR20547] (Abcam, ab213204, ChIP grade): [https://www.abcam.com/6x-his-tag-antibody-

epr20547-chip-grade-ab213204.html]
Anti-H3K9me3 (Diagenode, SN-146-100): [https://www.diagenode.com/en/p/h3k9me3-polyclonal-antibody-classic-100-ul]
Anti-SETDB1 (Bethyl, A300-121A): [https://www.thermofisher.com/antibody/product/SETDB1-Antibody-Polyclonal/A300-121A]
Anti-SETDB1 (Santa Cruz Biotechnology, ESET (G-4): sc-271488): [https://www.scbt.com/p/eset-antibody-g-4]
Anti-ZNF638/NP220 (Bethyl, A301-548A-M): [https://www.thermofisher.com/antibody/product/ZNF638-NP220-Antibody-Polyclonal/A301-548A]
Furthermore, these antibodies have been validated by IP, WB and IF in our lab.

Eukaryotic cell lines

Policy information about cell lines and Sex and Gender in Research

Cell line source(s)	Pooled human umbilical vein endothelial cells (HUVECs) were purchased from PromoCell (C-12203, Lot No. 405Z013, 408Z014, 416Z042, Heidelberg, Germany) and originate from umbilical cord/ umbilical vein of caucasians (405Z013: 2 males, 1 female; 408Z014: 2 males, 1 female; 416Z042: 2 males, 2 females). Human embryonic kidney 293 cells (HEK293) were from ATCC (Manassas, USA) and Lenti-X 293T cells from Takara (632180, Japan).
Authentication	None of the cell lines used were autheticated.<br>HUVECs were tested for endothelial cell marker expression, their angiogenic potential, their response to VEGF, to TNFa and to laminar shear stress. Transcriptomic analysis of the cells was also performed and compared to other published HUVEC data. HEK293 and Lenti-X 293T cells served for protein purification and virus production purposes.
Mycoplasma contamination	All cell lines were tested negative for Mycoplasma contamination.
Commonly misidentified lines<br>(See ICLAC register)	No commonly misidentified lines were used.
