# Supplementary material for: HIF1α-AS1 is a DNA:DNA:RNA triplex-forming lncRNA interacting with the HUSH complex
Source: Nat Commun. 2022 Nov 2;13:6563. doi: 10.1038/s41467-022-34252-2 (PMC9630315; doi:10.1038/s41467-022-34252-2)
Supplement: Supplementary file 10 — Source Data [file 41467_2022_34252_MOESM10_ESM.pdf]

Source Data file containing uncropped blots and gels from main figures

Figure 3a

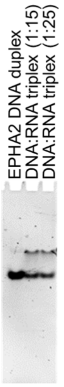

Figure 4c

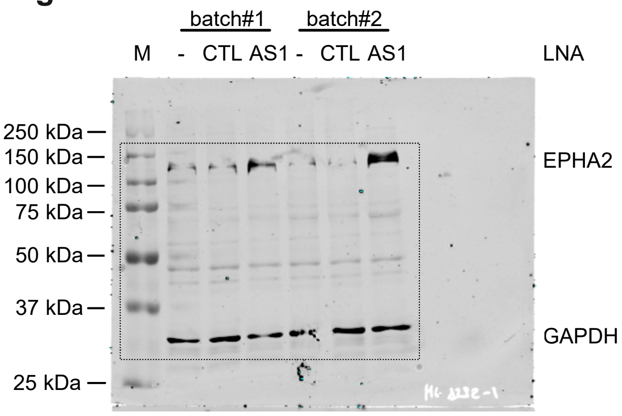

dashed line: figure shown in manuscript

Figure 4l

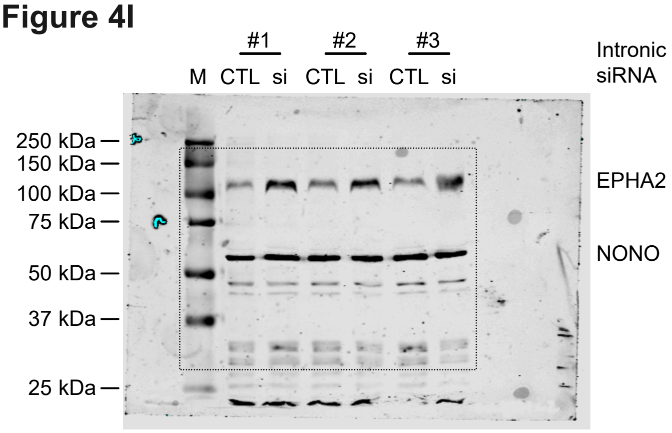

dashed line: figure shown in manuscript

Figure 4m

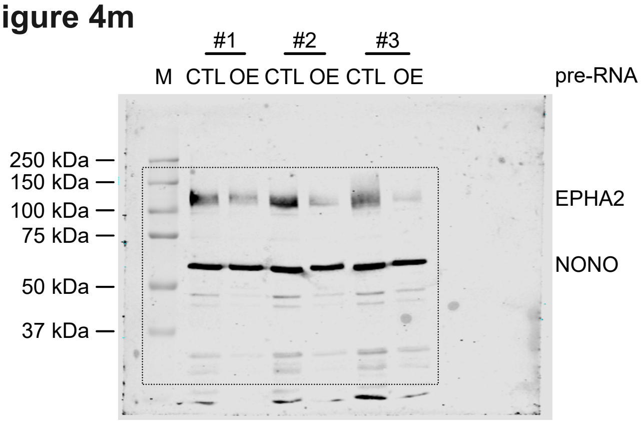

dashed line: figure shown in manuscript
